# Ageing as “early‐life inertia”: Disentangling life‐history trade‐offs along a lifetime of an individual

**DOI:** 10.1002/evl3.254

**Published:** 2021-09-08

**Authors:** Hanne Carlsson, Edward Ivimey‐Cook, Elizabeth M. L. Duxbury, Nathan Edden, Kris Sales, Alexei A. Maklakov

**Affiliations:** ^1^ School of Biological Sciences University of East Anglia Norwich NR4 7TJ United Kingdom

**Keywords:** Ageing, antagonistic pleiotropy, life‐history evolution, senescence

## Abstract

The theory that ageing evolves because of competitive resource allocation between the soma and the germline has been challenged by studies showing that somatic maintenance can be improved without impairing reproduction. However, it has been suggested that cost‐free improvement in somatic maintenance is possible only under a narrow range of benign conditions. Here, we show that experimental downregulation of insulin/IGF‐1 signaling (IIS) in *C. elegans* nematodes, a robustly reproducible life span‐ and health span‐extending treatment, reduces fitness in a complex variable environment when initiated during development but does not reduce fitness when initiated in adulthood. Thus, our results show that the costs and benefits of reduced IIS can be uncoupled when organisms inhabit variable environments, and, therefore, do not provide support for the resource allocation theory. Our findings support the theory that the force of natural selection on gene expression in evolutionarily conserved signaling pathways that shape life‐history traits declines after the onset of reproduction resulting in organismal senescence.

Ageing, or senescence, is a physiological deterioration of an organism resulting in reduced health, impaired reproduction, and increased probability of death with advancing age (Partridge and Barton 1993; Flatt and Partridge 2018). Although ageing is deleterious for evolutionary fitness of an organism (Bouwhuis et al. 2012; Nussey et al. 2013; Kowald and Kirkwood 2015; Gaillard and Lemaitre 2020), ageing can evolve because the force of natural selection on traits declines after reproductive maturity (Medawar 1952; Williams 1957; Hamilton 1966; Caswell and Shyu 2017). This can lead to the accumulation of deleterious mutations whose effects on fitness are concentrated in late life (hence, mutation accumulation theory [Medawar 1952]) or selection for alleles that increase fitness in early life at the expense of fitness in late life (hence, antagonistic pleiotropy theory [Williams 1957]). Finally, the “disposable soma” theory (DST), a physiological account of antagonistic pleiotropy, maintains that ageing results from competitive resource allocation between reproduction and somatic maintenance, leading to the slow gradual accumulation of unrepaired damage to the soma (Kirkwood 1977). The DST is supported by a body of empirical work suggesting that increased reproduction correlates with faster ageing (Kirkwood and Rose 1991; Lemaitre et al. 2015).

The last couple of decades have seen major discoveries in the biology of ageing suggesting that ageing is shaped by evolutionarily conserved and interconnected genetic pathways (insulin/insulin‐like signaling [IIS] and target‐of‐rapamycin [TOR]) that jointly regulate development, growth, reproduction, and longevity (Kenyon 2010; Gems and Partridge 2013; Flatt and Partridge 2018). Mutations in key elements of these pathways can result in life span increases (Kenyon 2010; Kenyon 2011), sometimes up to 100−500% (Chen et al. 2013), but are often associated with costs to other life‐history traits such as impaired development and reduced reproductive performance (Briga and Verhulst 2015; Maklakov et al. 2017). Thus, these studies are in line with both antagonistic pleiotropy and the DST. However, recent advances in this field suggest that increased somatic maintenance and longevity can be uncoupled from the detrimental effects on development and reproduction (reviewed in Flatt and Partridge 2018; Maklakov and Chapman 2019). The pioneering work by Dillin et al. (2002) in *C. elegans* nematode worms demonstrated that downregulation of IIS pathway during development increases life span and reduces reproduction; however, downregulation of IIS pathway after reproductive maturation achieves the same level of life span extension without reproductive costs. Further to this, a later study by Lind et al. (2019) showed that adulthood‐only downregulation of IIS can increase offspring quality, possibly by increasing parental investment into egg size. These results contradict the predictions of the DST and suggest the possibility of cost‐free improvement in survival by regulating gene expression in adulthood (Lind et al. 2021). However, it is possible that cost‐free life span extension is only possible in a narrow range of benign and stable laboratory conditions, whereas in complex and variable environments disabling IIS/TOR either will not increase life span (Van Voorhies et al. 2005) or will result in a net fitness cost. Building on the observation that IIS pathway is key for how an organism responds to a broad spectrum of environmental cues, Regan et al. (2020) suggested that disabling the IIS pathway in variable environments will reduce fitness. Indeed, the studies that “uncouple” improved somatic maintenance from impaired development and/or reproduction were conducted in benign stable environments.

A different proposition is the set of theories that we jointly refer to as “early‐life inertia” theories of ageing (E‐LIT). These theories are referred to in the literature as the developmental theory of ageing (de Magalhaes and Church 2005; de Magalhaes 2012; Maklakov and Chapman 2019; Lind et al. 2021), hyperfunction (Blagosklonny 2006, 2010; Gems and Partridge 2013; Ezcurra et al. 2018; Lind et al. 2019), or programmatic theory of ageing (de Magalhaes 2012; Gems and de Magalhães 2021) (Fig. 1). The E‐LIT posits that physiological processes are optimized for development and early‐life reproduction and can cause harm in old age but remain largely unopposed by natural selection because the force of selection on traits declines with age. The E‐LIT predicts that the costs and the benefits of IIS downregulation can be separated along the life course of an organism, such that postdevelopment downregulation can improve the soma without a cost to reproduction by abolishing “early‐life inertia” in the level of IIS signaling. Like the DST, the E‐LIT is a form of antagonistic pleiotropy (de Magalhaes and Church 2005; Blagosklonny 2006; Maklakov and Chapman 2019) (Fig. 1). Unlike the DST, this theory maintains that some aspects of gene expression are tuned to early‐life fitness and are insufficiently regulated in late life due to reduced force of selection. For example, high levels of IIS signaling promote development, growth, and reproduction but can generate damage late in life. Here, we directly test the predictions derived from the DST and the E‐LIT by downregulating IIS signaling in *Caenorhabditis elegans* nematodes using *daf‐2* RNA interference (RNAi) approach, starting either in early development or after reproductive maturity in a variable environment. We investigated the effects of age‐specific *daf‐2* RNAi on age‐specific reproduction, survival, health span (healthy life span), individual fitness, and offspring quality.

**Figure 1:**
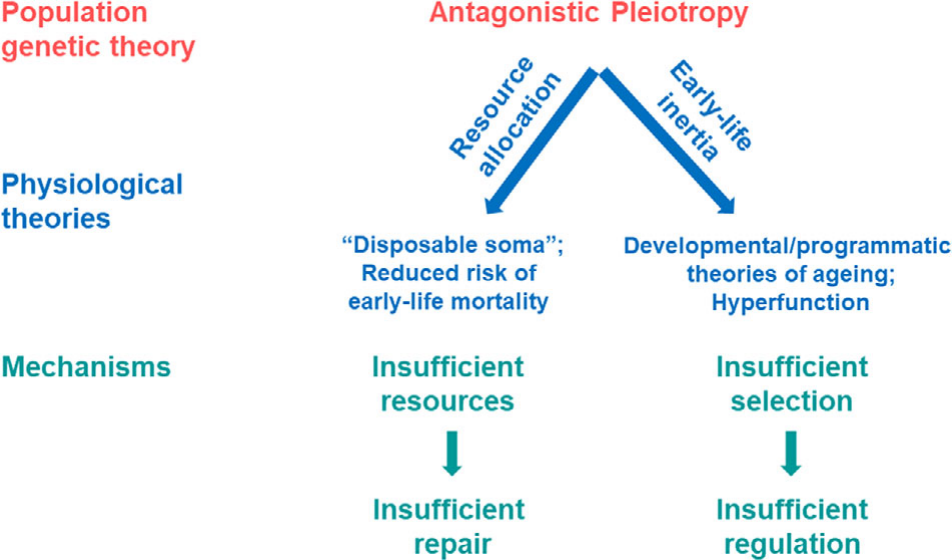
Antagonistic pleiotropy theory of ageing (AP) is a population genetic theory, which maintains that selection favors alleles that increase fitness in early life at the expense of fitness in late life because the force of selection on traits declines with age (Williams 1957). There are two main routes in which AP alleles can operate. First, AP alleles can control allocation of limited resources between life‐history traits. For example, the “disposable soma” theory (DST) (Kirkwood 1977), a physiological theory of ageing, is based on the putative trade‐off between resource allocation to growth, reproduction, and somatic maintenance. Under the DST, alleles that increase allocation to rapid growth and early‐life reproduction at the cost of late‐life survival and reproduction can be favored by selection. The recently proposed hypothesis that selection can favor increased early‐life survival at the cost of reduced late‐life survival is another special case of how AP can work via resource allocation (Omholt and Kirkwood 2021). All resource allocation versions of AP share the same underlying mechanism—insufficient resources that are optimally allocated across life‐history traits to maximize fitness, resulting in insufficient resources for somatic maintenance and repair. Second, AP alleles can cause late‐life damage by continuing to function in a way that was beneficial in early life but detrimental in an adult organism (“early‐life inertia” theories of ageing). For example, natural selection on gene expression is maximal in early life, from development until the age of first of reproduction, and is expected to decline after the onset of reproduction (Hamilton 1966). Insufficient selection on gene expression can result in suboptimal levels of gene expression in late life leading to senescence. There are several different hypotheses that have been proposed, for example, the developmental/programmatic theories of ageing (de Magalhaes and Church 2005; de Magalhaes 2012; Gems and de Magalhães 2021) and hyperfunction (Blagosklonny 2006, 2010). All these theories share the same underlying principle that natural selection optimizes organismal physiology for development and early‐life reproduction and fails to regulate late‐life performance (Maklakov and Chapman 2019).

## Results

We tested the age‐specific effects of *daf‐2* RNAi knockdown in three different ways. In Experiment A, we kept the animals in a variable environment (fluctuating temperature and light, see *Methods*) and exposed them to *daf‐2* RNAi/empty vector control from the egg stage. In Experiment B, we kept the animals in a variable environment from the egg stage and exposed them to *daf‐2* RNAi/empty vector from early adulthood. The direct comparison between Experiments A and B allows us to disentangle the effects of *daf‐2* knockdown during development and after reproductive maturity. Finally, in Experiment C we kept the animals in standard benign conditions during development and only introduced a variable environment and *daf‐2* RNAi from early adulthood. Thus, Experiment C specifically asks what would happen if we remove environmental variability in early life. This setup allows us to investigate the importance of developing in stable versus variable environments.

### GENE EXPRESSION ANALYSIS

**Figure 2:**
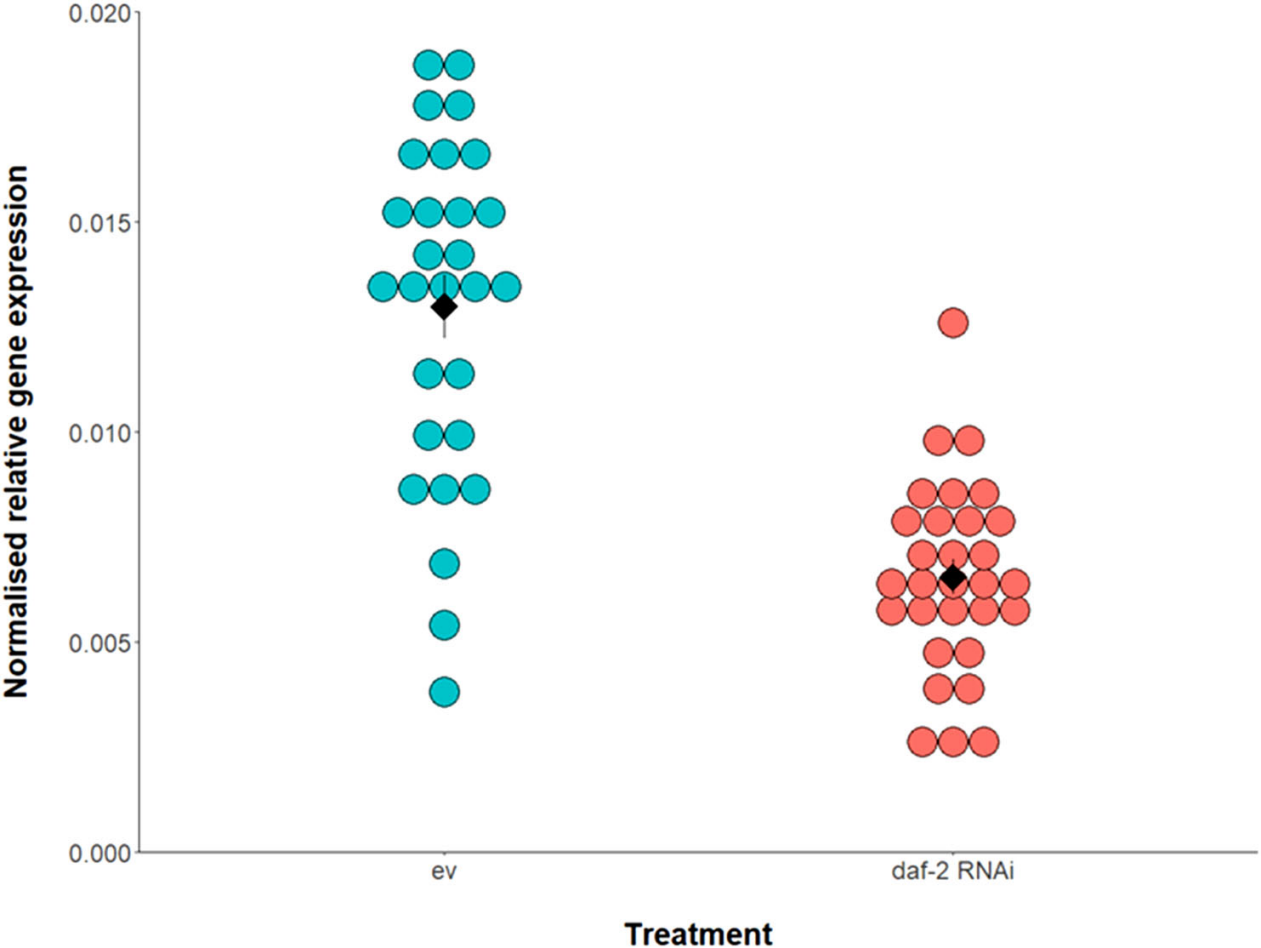
Normalized *daf‐2* expression following *daf‐2* RNAi treatment (blue) versus untreated empty vector controls (ev, orange). RNAi was delivered from the late‐L4 stage and gene expression was quantified in 2‐day old adults using qRT‐PCR, in individual worms (ev controls: *n* = 29; *daf‐2* RNAi: *n* = 30; as separate points). Arithmetic mean of biological replicates shown as a black diamond with ± 1 standard error bars. Normalized *daf‐2* expression (2^–Δ^ *^Ct^*) was calculated relative to expression of the *actin‐3* reference gene (Schmittgen and Livak 2008).

We confirmed, using quantitative reverse transcriptase polymerase chain reaction (qRT‐PCR) analysis, that feeding nematodes bacteria that expresses double‐stranded RNA for *daf‐2* downregulated *daf‐2* gene expression by 51% in 2‐day old adults, compared with daf‐2 expression in untreated age‐matched controls (based on gene expression fold change, 2^–ΔΔ^ *^Ct^*). This represented a significant downregulation in the relative gene expression of *daf‐2* via RNAi (Table S8; Fig. 2).

Technical replicates were highly repeatable for all samples (coefficient of variation [CV] < 1.5% for all except three samples that were CV < 2.8%), confirming the repeatability of the qRT‐PCR assay. Biological replicates showed considerably more variation in relative gene expression within each RNAi treatment (controls: CV = 9%, *daf‐2* RNAi: CV = 8%; Fig. 2), but within the expected range for *C. elegans* based on previous qRT‐PCR expression analyses for different genes between pooled samples (Lind et al. 2021) and between individual worms (Chauve et al. 2020).

### PARENTAL GENERATION

**Figure 3:**
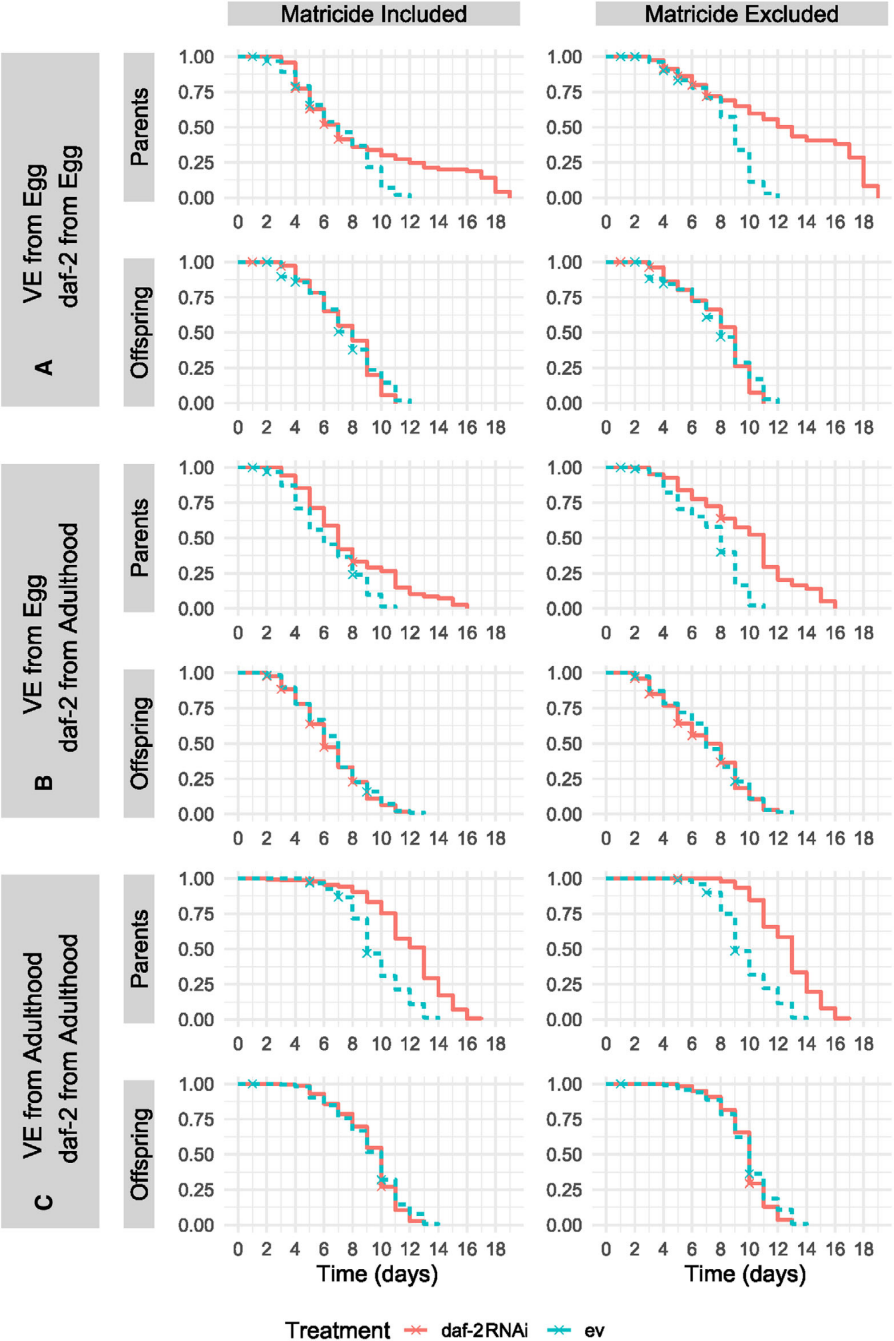
Survival curves for parental and offspring generations across three experiments. Nematodes are either in a variable environment (VE) and exposed to *daf‐2* RNAi/empty vector (ev) from the egg (Experiment A); in a variable environment from the egg and exposed to *daf‐2* RNAi/empty vector from early adulthood (Experiment B); or in standard benign conditions during development and in variable environment and *daf‐2* RNAi/empty vector from early adulthood (Experiment C). All deaths are presented in the left‐column plots, whereas matricidal deaths are censored in the right‐column plots. *daf‐2* RNAi treatment increased parental survival across all treatments regardless of matricide (*Log‐Odds* with/without Matricide: A = −0.63/−1.57; B = −0.95/−1.75, C = −2.08/−3.09; all *P* < 0.001), whereas there was no effect on the offspring (*Log‐Odds* with/without Matricide: A = 0.07/0.10; B = 0.10/0.05; C = 0.10/0.17; all *P* > 0.05).

**Figure 4:**
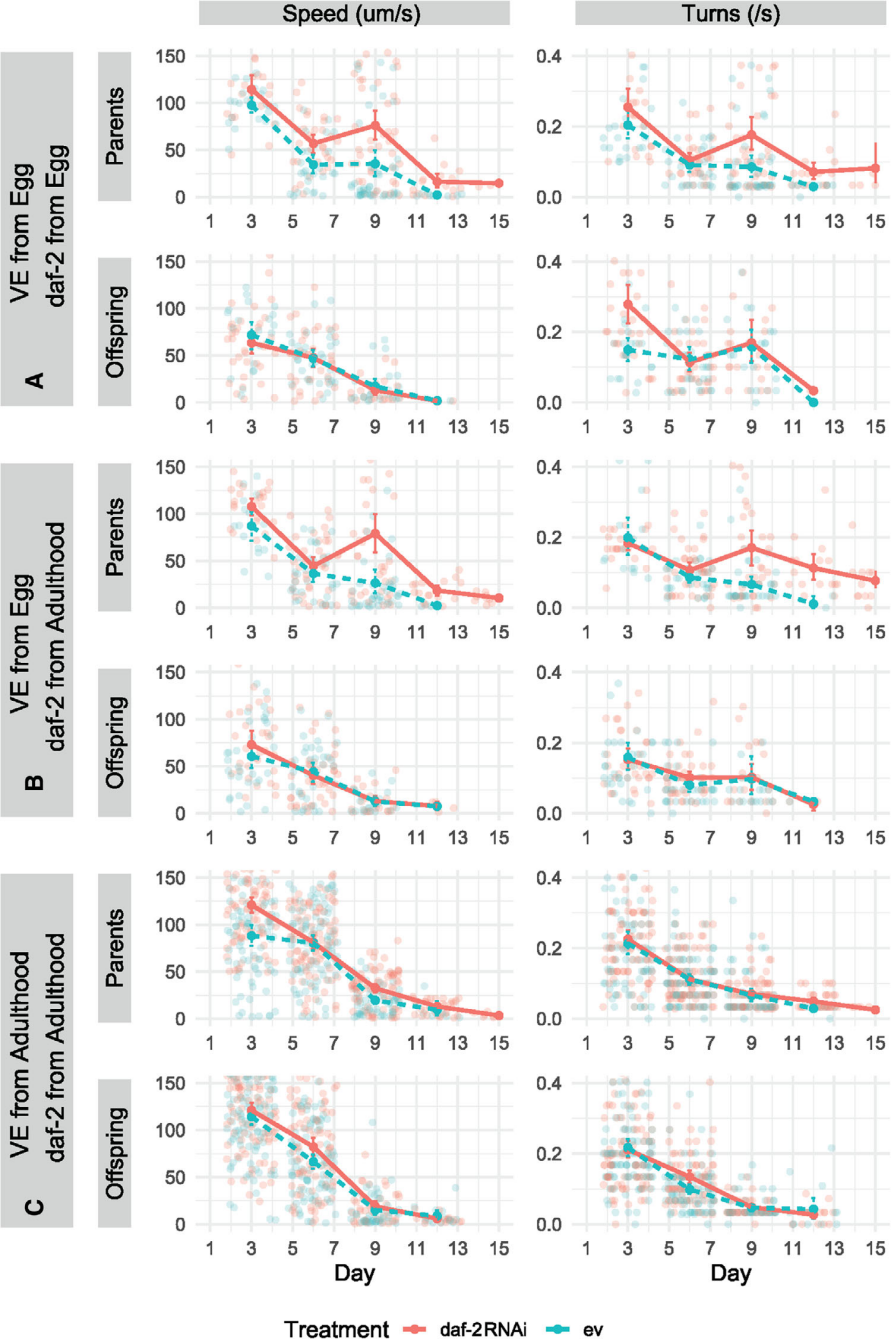
Health span metrics for parental and offspring generations across three experiments (see Fig. 1 legend for details). Speed (movement/s) in the left‐column plots and body bends (turns/s) in the right‐column plots. *daf‐2* RNAi treatment improved parental health span in Experiments A and B (Speed:Day/Turns:Day: A = 0.19 [0.05], *P* < 0.001/0.10 [0.04], *P* = 0.011; B = 0.18 [0.05], *P* < 0.001/0.17 [0.04], *P* < 0.001), but not in Experiment C (−0.04 [0.03], *P* = 0.094/0.01 [0.02], *P* = 0.59). There was no effect on the offspring (A = 0.04 [0.06]/−0.07 [0.05]; B = −0.04 [0.06]/0.02 [0.04]; C = −0.00 [0.03]/0.02 [0.02]; all *P* > 0.05).

**Figure 5:**
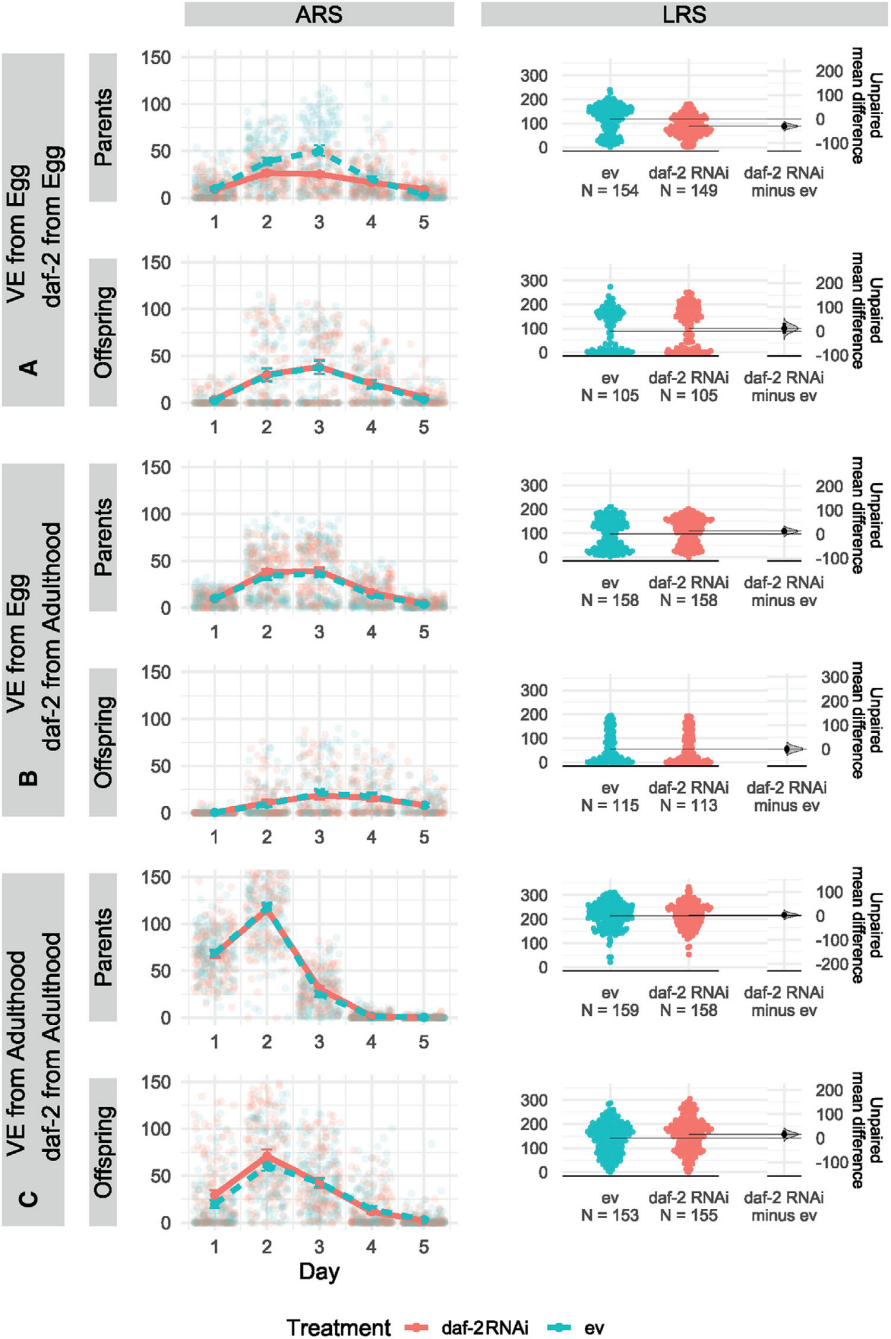
Age‐specific reproductive success (ARS, left‐column plots) and lifetime reproduction success (LRS, right‐column plots) for parental and offspring generations across three experiments (see Fig. 1 legend for details). We plotted raw data points and means ± SE for ARS (ggplot in R) and raw data points together with the difference in effect size based on nonparametric bootstrapping for LRS (dabestr package in R). In the parental generation, *daf‐2* RNAi treatment reduced ARS (Treatment:Day = −1.49 [0.16], *P* *< *0.001) and LRS (−0.29 [0.05], *P* < 0.001) in Experiment A but had no effect on reproduction in Experiments B (Treatment:Day = 0.07 [0.15], *P* = 0.635) and C (Treatment:Day = 0.13 [0.17], *P* = 0.459). There was little evidence for parental *daf‐2* RNAi effect on offspring ARS and LRS, although there was small positive effect in Experiment C (0.11 [0.04], *P* = 0.013).

*Caenorhabditis elegans* fed with bacteria with *daf‐2* RNAi construct had significantly reduced mortality risk, and thus increased life span, in all three of the experimental environments (A, B, C median life spans/days = empty vector: 9, 8, 9, and daf‐2: 12, 10.5, 13; Fig. 3; Tables 1 and S1A–F). The greatest difference in median life span between individuals fed empty vector and *daf‐2* RNAi was in experiment C (variable environment and *daf‐2* RNAi from adulthood; Fig. 3). Although *daf‐2* RNAi individuals in both experiments A and B exhibited a corresponding increase in health span (in both of the measured health span metrics), experiment C individuals, despite displaying the largest increase in median longevity, failed to exhibit a similar pattern (Fig. 4; Tables 1, S5A–C, and S6A–C). For reproduction, Experiment A individuals, which were exposed to a variable environment and *daf‐2* RNAi from the egg stage, exhibited decreased age‐specific reproductive success (ARS), lifetime reproductive success (LRS), and individual fitness (λ_ind_) (Fig. 5; Tables 1, S2A, S2B, S3A, S3B, and S4A). For experiment B and C individuals, no detectable differences in fitness were identified (Fig. 5; Tables 1, S2C–H, S3C–H, S4B, and S4C).

**Table 1:** Summary of the key results

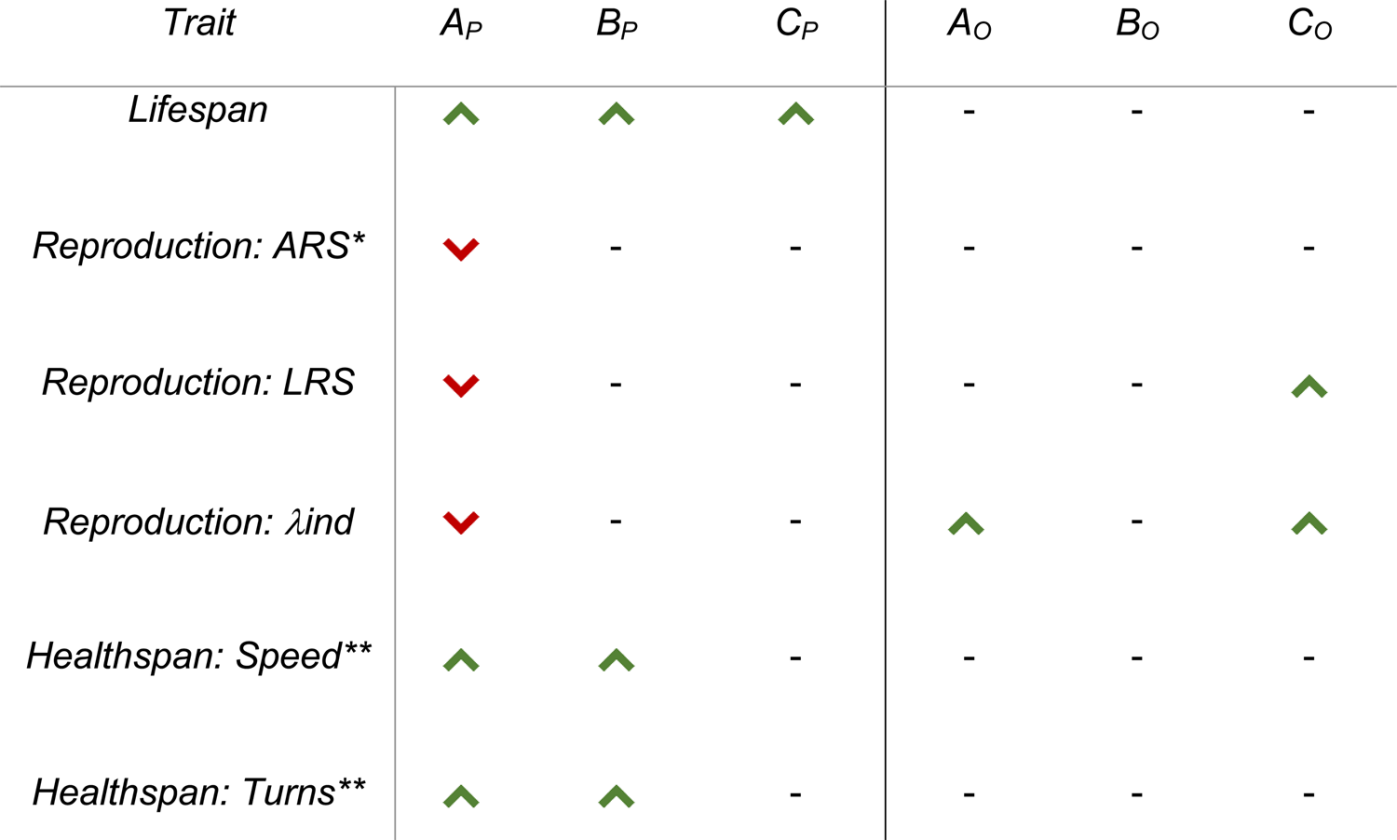

The effect of *daf‐2* RNAi on various measured traits in relation to empty vector in either the parent (_P_) or offspring (_O_) generation of experiments A (variable environment and *daf‐2* RNAi from egg), B (variable environment from egg, *daf‐2* RNAi from adulthood), and C (variable environment and *daf‐2* RNAi from adulthood). Upward‐facing arrows (green) denote a significant positive effect (α = 0.05), downward‐facing (red) denote a significant negative effect, and a dash denotes no detectable effect of *daf‐2* RNAi.

^*^The direction of arrow shown here denotes an interaction with treatment and both the quadratic and linear forms of time.

^**^The direction of arrow shown here denotes an interaction with treatment and the linear form of time.

### OFFSPRING GENERATION

Parental *daf‐2* RNAi had no effect on offspring survival across all three experiments (A, B, C median life spans/days = empty vector: 8, 7, 10, and daf‐2: 8, 6, 10; Fig. 2; Tables 1 and S1G–L). Similarly, there was no effect on health span metrics in all three experiments (Fig. 3; Tables 1, S5D–F, and S6D–F). However, parental *daf‐2* RNAi increased offspring LRS in Experiment C and λ_ind_ in both Experiments A and C (Fig. 4; Tables 1, S2I–N, S3I–L, and S4D–F). Because the differences on offspring number were only evident during the first 2 days of reproduction, this did not result in detectable differences in ARS between treatments.

## Discussion

Our findings support the view that age‐specific life histories are shaped by evolutionarily conserved genetic pathways, such as the IIS pathway, and that selection on the expression of genes involved in these pathways declines after the onset of reproduction. Specifically, we found that adulthood‐only downregulation of IIS improves both life span and health span in variable and temporally changing environments without any negative effects on individual fitness and offspring quality. On the other hand, when IIS is downregulated during development, the animals pay a price in reduced reproduction and individual fitness. Thus, IIS regulates fitness and ageing independently. These results contradict the hypothesis that ageing results primarily from slow gradual accumulation of unrepaired somatic damage because of competitive resource allocation between damage repair and reproduction. Crucially, they do not support the hypothesis that downregulation of IIS pathway will reduce fitness in variable environments. Recently, it has been suggested that *C. elegans* may vent yolk to provide extra food for their offspring and that *daf‐2* mutants lack this ability (Kern et al. 2020). However, our data suggest that fitness of offspring of *daf‐2* RNAi parents was not impaired in any way and, in some cases, even improved. Instead, our results support the hypothesis that ageing is accelerated by unnecessarily high levels of nutrient‐sensing signaling in adulthood. Consequently, postdevelopment downregulation of IIS improves survival and ability to move in old age in variable environments.

The effect size of life span extension via *daf‐2* knockdown in a variable environment was considerably smaller than in previously reported experiments in benign stable environments (Dillin et al. 2002; Lind et al. 2019). This was particularly true when matricidal deaths were not censored. Although such censoring is habitual in biogerontology, we believe it is very important to include all deaths when trying to understand the evolutionary consequences of any intervention aimed at life span extension. In our hands, *daf‐2* RNAi *C. elegans* N2 can survive up to 57 days in benign environments compared to 22 days on empty vector (see Lind et al. 2019), whereas in this study the differences in maximum longevity were 19 versus 12 days, 16 versus 11 days, and 17 versus 14 days, across three different experiments. Overall, adulthood‐only *daf‐2* RNAi does improve survival and, importantly, locomotory performance, but the fitness benefits are relatively minor. We previously showed that parental *daf‐2* RNAi can increase offspring fitness in benign environments (Lind et al. 2019). We found a similar effect in this study when animals were kept in benign environments in early life and transferred to variable environment in adulthood. However, when animals instead developed in a variable environment such positive parental effects largely disappeared. The best‐fitting model suggests that λ_ind_ was increased in offspring of *daf‐2* RNAi parents in Experiment A. However, the effect size is very small and not supported by bootstrapping analyses (see Fig. 4), so this result should be viewed with caution. It could offset some of the costs of early *daf‐2* RNAi but is unlikely to play a major role given the large effect size reduction in ARS, LRS, and λ_ind_ of *daf‐2* RNAi parents in Experiment A. Notably, we did not detect strong improvements in parental fitness in our study because survival benefits occurred during the postreproductive period. It is possible, however, that adulthood *daf‐2* RNAi worms would be more resistant to some other environmental stresses, which may improve survival during the crucial days of reproduction and, therefore, improve fitness. Furthermore, improved locomotion of *daf‐2* RNAi animals could be beneficial in escaping predation, unfavorable environmental conditions, and while searching for food. Future work is necessary to establish this, but such putative fitness benefits would be in line with antagonistically pleiotropic function of *daf‐2*.

The antagonistic pleiotropy theory of ageing is a broad concept that covers different types of alleles that can confer an early‐life advantage at the cost of reduced performance in late life (Williams 1957). Much of the empirical research have centered around damage accumulation as a result of imperfect repair of the soma. However, one of the straightforward yet least studied mechanisms of ageing by antagonistic pleiotropy is the deleterious effects of the ontogenetic patterns of gene expression that persist into adulthood (de Magalhaes and Church 2005; Blagosklonny 2006; de Magalhaes 2012; Ezcurra et al. 2018; Lind et al. 2019; Maklakov and Chapman 2019), because they are insufficiently opposed by declining force of natural selection with age (Williams 1957; Hamilton 1966). This hypothesis was first put forward in the seminal paper by Williams (1957) who described how the same function of an allele can have different effects on fitness during the life course of the organism. Since then, this approach has been advanced further by several authors upon the accumulation of the new data (de Magalhaes and Church 2005; Blagosklonny 2006; Blagosklonny 2008; de Magalhaes 2012; Gems and Partridge 2013; Ezcurra et al. 2018; Lind et al. 2019; Maklakov and Chapman 2019; Lind et al. 2021). All these theories share the core concept that weak late‐life selection fails to prevent “early‐life inertia,” where physiological processes that are optimized for successful early‐life reproduction (including, but not limited to, development) start causing harm with advancing age.

Interestingly, although the traditional approach in evolutionary biology of ageing maintained that ageing results from a large number of mutations with small deleterious effects in late life, the emerging view is that ageing is instead shaped by evolutionary conserved genetic pathways that modulate key life‐history traits, such as development, reproduction, and survival (Fontana et al. 2010; Kenyon 2010; Flatt and Partridge 2018). Therefore, genetic or environmental modulation of the expression of a small number of key upstream genes in such pathways can result in a major change of the individual life history. When such a change in life history in the direction of increased somatic preservation occurs before reproductive maturity, it results in reduced reproduction and individual fitness. However, these costs can be avoided by postponing the change in life history until after the reproductive maturity. Our experimental findings support this view and suggest that IIS can be modulated in adult organisms to improve life span and health span in variable environments. Further research is now required to establish the generality of these findings across different environments in different taxa. We envisage at least three promising research directions. First, we need to broaden the environmental complexity to match natural environments more closely by introducing pathogens, predators, and ever more natural abiotic conditions. Second, age‐specific manipulation of gene expression is becoming increasingly available in different organisms (e.g., nematodes, flies, Daphnia, beetles), which will allow testing these ideas in a broad range of taxa, which is crucial. Third, the development of more precise “biological‐age clocks” may allow testing the effect of “early‐life inertia” on ageing in the long‐lived organisms, such as fish, reptiles, birds, and mammals, in their natural habitats.

## Methods

The data uploaded to Dryad: https://doi.org/10.5061/dryad.f7m0cfxwz.

### STRAIN

We used N2 (Bristol) strain of *C. elegans* obtained from Caenorhabditis Genetics Centre in all experiments. After defrosting, the worm cultures were maintained for two generations to build a healthy population and clean the strain from potential bacterial contamination through bleaching. During recovery from freezing and up until the start of the experiment, worms were fed *Escherichia coli* of the strain OP50‐1 (pUC4K) from J. Ewbank, Centre d'Immunologie de Marseille‐Luminy, France, seeded onto NGM plates containing Streptomycin (100 μg/ml), Ampicillin (100 μg/ml), and Nystatin (10 μg/ml).

### RNAi

The nematodes were fed an RNAi clone of *E. coli* HT115 (DE3), transformed using the pL4440 vector with an insert expressing dsRNA corresponding to the *C. elegans daf‐2* gene. The clone was obtained from the Vidal library. As a negative control, we used the same *E.coli* HT115 (DE3) clone carrying a pL4440 vector without an insert (empty vector, EV). RNAi clones were cultured in LB medium with the addition of 50 μg/ml Ampicillin to ensure the retention of the vector. These cultures were seeded onto 35‐mm standard NGM plates containing 100 μg/ml Ampicillin, as well as the fungicide Nystatin (10 μg/ml) and IPTG (1 mM), required for the transcription of the *daf‐2* RNAi insert. Seeded plates were left to grow in 20°C overnight to produce an ad libitum food source for the nematodes.

### VALIDATION OF DAF‐2 GENE EXPRESSION UNDER RNAI

We quantified the extent of downregulation of *daf‐2* following feeding RNAi, using qRT‐PCR. First, we set up a separate batch of worms (from the same frozen population of N2) identically to those in the main experiment and RNAi was applied from late‐L4 stage. All worms for the gene expression assay were maintained under standard conditions of 20°C and constant darkness.

Worms were collected on day 2 of adulthood to assay the downregulation of *daf‐2* in individuals at peak reproduction (final sample size of 30 worms on *daf‐2* RNAi and 29 worms on empty vector control *E. coli*). To do this, we picked individual worms onto unseeded plates and allowed them to crawl around to remove surface bacteria and separate day 2 adults from their eggs (Chauve et al. 2020), and to ensure ingested bacteria was excreted (Ghafouri and McGhee 2007).

Individual cleaned worms were transferred into 10 μl of worm lysis buffer (containing 1:100 diluted DNase; both from Ambion Power SYBR Green Cells‐to‐Ct kit) in the separate domed lids of 0.2‐ml PCR tubes, immediately spun down into the bottom of the PCR tubes, and flash frozen in liquid nitrogen (Chauve et al. 2020). To crack the tough nematode cuticle and release nucleic acids, we performed 10 freeze‐thaw cycles, by transferring the PCR tubes between liquid nitrogen and a 40°C temperature, before homogenizing the samples in a thermal mixer (Eppendorf ThermoMixer C) set at 4°C, for 30 min at 1800 rpm (Chauve et al. 2020). We confirmed, using Nanodrop spectrometry (Thermo Scientific), that it was possible to obtain ∼30–55 ng of RNA from our single worm samples (Ly et al. 2015; Chauve et al. 2020).

We reverse transcribed DNase‐treated RNA using the Ambion Power SYBR Green Cells‐to‐Ct kit, following the manufacturer protocol. We included a no reverse transcriptase control (NRTC) per RNAi treatment, for which the RT enzyme was substituted with nuclease‐free water. The synthesized cDNA was used undiluted for PCR and qRT‐PCR.

To confirm that any contaminating genomic DNA had been removed, we performed a standard PCR with a 10 μl reaction and an annealing temperature of 60°C. We ran 5 μl of the PCR reaction on a 1% agarose gel using ethidium bromide and confirmed both the absence of amplification in the NRTCs verifying the successful removal of any contaminating gDNA and also the successful amplification of cDNA for primer pairs.

The qRT‐PCR was performed on an Applied Biosystems 7500 Real‐Time PCR System using the Power SYBR Green Cells‐to‐Ct kit (Ambion) with the following PCR cycle: 95°C for 10 min, followed by 40 cycles of 95°C for 15 s and 60°C for 1 min. The total reaction volume was 20 μl of which 4 μl was cDNA. We used primers specific for the target gene of interest (*daf‐2*) and for a reference gene—the housekeeping gene, *actin‐3* (T04C12.4), commonly used for *C. elegans* (Akay et al. 2017). Primer sequences are listed in Table S7. Primers were designed based on MIQUE guidelines (Bustin et al. 2009) and taken from (Chauve et al. 2020) for *daf‐2* and (Akay et al. 2017) for *actin‐3*.

Two qRT‐PCR reactions (technical replicates) were carried out per sample per primer pair, to check for repeatability and RNAi treatments were split evenly across the three plates, to control for any minimal plate effects. We also included two negative template controls (nuclease‐free water substituted for cDNA) and one NRTC per primer pair per plate, to test for any contamination.

To calculate relative gene expression, we determined Δ*Ct* as the difference between the qRT‐PCR cycle thresholds (*Ct* values) of the target gene of interest (*daf‐2*) and the reference gene, for each sample. The arithmetic mean of the *Ct* values for the two technical replicates per gene, per sample was used in Δ*Ct* calculations. Statistical analyses were performed on Δ*Ct* (Chauve et al. 2020), using a linear model with Gaussian error structure, to determine the effect of RNAi treatment (*daf‐2* RNAi vs. empty vector controls), qRT‐PCR plate, and their interaction on relative gene expression. The Shapiro‐Wilk's normality test confirmed that Δ*Ct* values satisfied the normality assumption of the linear models (*W* = 0.968, *P* = 0.121).

CV (%) in Δ*Ct* between biological replicates each RNAi treatment, and in *Ct* values between technical replicates per gene, per sample, was calculated as the standard deviation divided by the mean for each comparison (Pfaffl 2001; Chauve et al. 2020), to determine biological variation in relative gene expression between individual worms and repeatability of the qPCR results, respectively.

To quantify fold change in gene expression (2^–ΔΔ^ *^Ct^*), we calculated the difference in the relative levels of mRNA for the target gene of interest compared to the reference gene (Δ*Ct*) between untreated controls and RNAi‐treated samples, using mean Δ*Ct* across all biological replicates per RNAi treatment (Schmittgen and Livak 2008).

### VARIABLE ENVIRONMENT

To create the variable environment, we programmed a climatic growth chamber (model MLR‐352H‐PE) for perpetual 24‐h cycles of temperature and light designed to be within the viable temperature range of this strain: (cycle: 6 h, 15°C, darkness; 6 h, 20°C, one light source ≈ 2000 Lux; 6 h, 25°C, three light sources ≈ 7000 Lux; 6 h: 20°C, one light source ≈ 2000 Lux). We chose this range of temperatures and light conditions as representative of a warm summer day in England (N2 strain is from Bristol) and we know that *C. elegans* can survive and reproduce at both the minimum and maximum points along this temperature range. We also know that daylight is a natural stress for nematodes (Muntane et al. 2018). We turned off the humidity control creating spontaneous humidity fluctuations between 20% and 80% relative humidity. All daily transfers of the worms outside the climatic chamber were performed during hours when light and temperature conditions most closely resembled those inside the chamber. Inside the chamber, the plates were positioned on a transparent glass shelf ensuring equal light exposure for every plate.

### REPRODUCTION AND LIFE SPAN ASSAYS

The parental generation (P0) was produced by age‐synchronized hermaphrodites at reproductive peak (day 2 of adulthood). The larvae were reared in groups for 3 days until transfer as L4 larvae onto individual plates. We used 40 individual worm‐plates per each of the two treatments within each block. We run four separate chronologically randomized blocks per each experiment.

The offspring generation (F1) was produced in a similar fashion following 4‐h egg‐laying by P0 worms from experimental treatments at their reproductive peak. Three eggs were then picked from each of the egg‐laying plates, keeping the number of siblings in a cohort the same across treatments. The peak reproduction was on day 3 when P0 worms were kept in a variable environment from egg stage (Experiments A and B, please see above), and at day 2 of adulthood when P0 worms were kept in the variable environment from L4 (Experiment C). Offspring developed together with siblings for 3 days at which point we picked one larva per parent. To capture any possible treatment‐induced variation in offspring developmental time, the age‐synchronized offspring were picked at a preset time at random with regard to their size and developmental stage.

During reproduction, both the parental and offspring generation were kept on individual plates and transferred every 24 h to record their daily egg production. These eggs were allowed to hatch into larvae and grow in 20°C for 2 days before counting live offspring. For logistical reasons, after reproduction had ceased at day 8 of adulthood, we placed the worms in small groups of maximum 10 per plate. The worms were observed and transferred daily onto fresh plates until death. We recorded separately which individuals died via matricide (internal hatching of offspring).

All reproduction and life span assays were fully blinded with respect to treatments.

### HEALTH SPAN ASSAYS

We used two metrics of body movement as proxies for health span: speed (track length including both forward and backward movement per second) and turns (body bends per second). Worms from both P0 and F1 generations were kept in groups of 10 individuals on three 35‐mm NGM plates per generation, treatment, and block (two blocks per treatment). Worms were transferred daily and then every third day starting on day 3 of adulthood. We recorded a 30‐s video of each plate using LAS V4.13 with the MultiTime video plugin, connected to a Leica MC170 HD camera. Worm movement was analyzed in the WormLab software version 2019.1.1 (MBF Bioscience, Williston, VT, USA).

### STATISTICAL ANALYSES OF LIFE SPAN, HEALTH SPAN, AND REPRODUCTION

All analyses were performed in R version 4.0.3 (Team 2016) using glmmTMB version 1.02.1 (Brooks et al. 2017; Magnusson et al. 2019) and visualized using ggplot2 version 3.3.3 (Wickham 2009) and dabestr version 0.3.0 (Ho et al. 2019). Lastly, model diagnostics were performed using DHARMa version 0.3.3.0 (Hartig 2020). Data were subset by experiment (A, B, or C) and by generation (Parent or Offspring) to determine the relative difference between *daf‐2* RNAi and empty vector within each generation and experimental treatment. Therefore, in all cases, the fixed effect of “Treatment” (daf‐2 RNAi or empty vector) was added as the primary explanatory variable in each model.

For survival, data were visualized both with and without the presence of matricide (the internal hatching of eggs, where ultimately the mother is consumed by her offspring). When matricide was included (see Fig. 2), individuals were not censored as the event of matricide had been observed (and was therefore given a “1” at death during analysis). To model survival, we used an event history analysis, which is qualitatively similar to a Cox Proportional Hazards model but has the advantage of not having to conform to the assumption of proportional hazards. We therefore modeled the probability of death per day (or mortality risk) using a binomial distribution where individuals were observed daily and given a “0” (denoting an alive or censored) or a “1” (denoting a death status). As individuals were repeatedly sampled across their life spans, a random effect of Individual ID was added. Experimental Block was added as a fixed effect to the model as it contained less than five levels that precluded inclusion as a random effect. Lastly, Set (the position within the climate chamber) was added as a random effect.

There were three distinct measures of fitness that were analyzed. For both ARS and LRS, an initial Poisson model with and without an observation‐level random effect were fitted. In each case, residuals were simulated (using the DHARMa package) and checked for overdispersion and evidence of zero‐inflation (see Ivimey‐Cook et al. 2021). If no overdispersion or zero‐inflation was detected, then the best fitting model from the two initial models was chosen by comparing Akaike's information criterion (AIC). However, if significant overdispersion or zero‐inflation was present, additional zero‐inflation components were fit to the two initial Poisson models and also to models fit with either Conway‐Maxwell Poisson (for LRS only), Negative Binomial (for ARS only), or Generalized Poisson (for ARS only) distributions. The best fitting model was subsequently chosen, by comparing model‐specific AIC (Nota bene [N.B.] the most parsimonious model within Δ6 AIC of the top model was selected [Richards 2007]) and accompanying measures of residual dispersion and zero‐inflation (identified again using the DHARMa package). If the level of under‐ or overdispersion was still significant, then additional dispersion parameters were added and models were compared again with AIC. In some cases (ARS for parents, ARS for offspring in Experiments A and B), the data were underdispersed after accounting for zero‐inflation and dispersion, suggesting that our results for these particular traits are relatively conservative (Harris et al. 2012; Brooks et al. 2019). For ARS, the linear and quadratic covariates of “Day” were added (N.B. as reproduction had ceased by day 6, only days 1–5 were used), as well as the higher order interaction with “Treatment” and an additional fixed effect of “Experimental Block.” Again, as individuals were repeatedly sampled, “Individual ID” was nested within “Set.”

For both LRS and individual fitness (λ_ind_), the latter given as the dominant eigenvalue from an age‐structured Leslie Matrix (Leslie 1945) and calculated using the popbio package version 2.7 (Stubben and Milligan 2007), only the fixed effects of “Treatment“ and “Experimental Block” were added, with a single random effect of “Set.” Lastly, for λ_ind_, a normal Gaussian distribution was used. For health span, two distinct measures were used. In each case, the dataset was trimmed to only include worms where the analyzed track duration was >2 s to reduce the chance of spurious measurements. The first, peristaltic speed, was a product of the total distance travelled and the duration of measurement. The second was a total count of the number of turns made by a worm during tracking. For both health span measures, models contained the fixed effects of “Treatment,” “Day,” and the subsequent higher order interaction along with the fixed effect of “Experimental Block.” Random effects of “Individual ID” nested within “Plate” were also added to account for both pseudoreplication and repeated measuring. The quadratic form of “Day” was initially considered but was rejected after visual inspection of the age‐specific data. In addition, as the number of turns was dependent on the total measurement length, a further covariate of “Track Duration” was added as a model offset to account for unequal sampling duration. Both measures included the same random effect structure as the ARS model. Lastly, although speed was fitted with a log‐normal distribution, turn count was fit following the same model procedure as the LRS model above.

## AUTHOR CONTRIBUTIONS

AAM conceived the study and developed it together with HC and EIC. HC, EMLD, NE, and KS collected the data. EIC, HC, and EMLD analyzed the data. AAM, EIC, HC, and EMLD wrote the draft. All authors commented on the manuscript.

## DATA ARCHIVING

All data will be made available on Dryad.

Associate Editor: A. Charmantier

## Supporting information

**Table S1A**. Full model output from the event history analysis excluding matricide for parents in experiment A.
**Table S1B**. Full model output from the event history analysis including matricide for parents in experiment A.
**Table S1C**. Full model output from the event history analysis excluding matricide for parents in experiment B.
**Table S1D**. Full model output from the event history analysis including matricide for parents in experiment B.
**Table S1E**. Full model output from the event history analysis excluding matricide for parents in experiment C.
**Table S1F**. Full model output from the event history analysis including matricide for parents in experiment C.
**Table S1G**. Full model output from the event history analysis excluding matricide for offspring in experiment A.
**Table S1H**. Full model output from the event history analysis including matricide for offspring in experiment A.
**Table S1I**. Full model output from the event history analysis excluding matricide for offspring in experiment B.
**Table S1J**. Full model output from the event history analysis including matricide for offspring in experiment B.
**Table S1K**. Full model output from the event history analysis excluding matricide for offspring in experiment C.
**Table S1L**. Full model output from the event history analysis including matricide for offspring in experiment C.
**Table S2A**. Model selection for ARS for parents in experiment A.
**Table S2B**. Summary table for the best identified ARS model for parents in experiment A.
**Table S2C**. Model selection for ARS for parents in experiment B. Showing the top three models in order of AIC (see the starting of the Supporting Information for more information).
**Table S2D**. Additional model selection for ARS for parents in experiment B. Showing the top three models (with the zero‐inflation components from above) with additional dispersion parameters shown in order of AIC (see the starting of the Supporting Information for more information).
**Table S2E**. Summary table for the best identified ARS model for parents in experiment B.
**Table S2F**. Model selection for ARS for parents in experiment C. Showing the top three models in order of AIC (see the starting of the Supporting Information for more information)
**Table S2G**. Additional model selection for ARS for parents in experiment C. Showing the top two models (with the zero‐inflation components from above) with additional dispersion parameters shown in order of AIC (see the starting of the Supporting Information for more information).
**Table S2H**. Summary table for the best identified ARS model for parents in experiment C.
**Table S2I**. Model selection for ARS for offspring in experiment A. Showing the top three models in order of AIC (see the starting of the Supporting Information for more information).
**Table S2J**. Summary table for the best identified ARS model for offspring in experiment A.
**Table S2K**. Model selection for ARS for offspring in experiment B. Showing the top three models in order of AIC (see the starting of the Supporting Information for more information).
**Table S2L**. Summary table for the best identified ARS model for offspring in experiment B.
**Table S2M**. Model selection for ARS for offspring in experiment C. Showing the top three models in order of AIC (see the starting of the Supporting Information for more information).
**Table S2N**. Summary table for the best identified ARS model for offspring in experiment C.
**Table S3A**. Model selection for LRS for parents in experiment A. Showing the top three models in order of AIC (see the starting of the Supporting Information for more information).
**Table S3B**. Summary table for the best identified LRS model for parents in experiment A.
**Table S3C**. Model selection for LRS for parents in experiment B. Showing the top three models in order of AIC (see the starting of the Supporting Information for more information).
**Table S3D**. Additional model selection for LRS for parents in experiment B. Showing the top three models (with the zero‐inflation components from above) with additional dispersion parameters shown in order of AIC (see the starting of the Supporting Information for more information).
**Table S3E**. Summary table for the best identified LRS model for parents in experiment B.
**Table S3F**. Model selection for LRS for parents in experiment C. Showing the top three models in order of AIC (see the starting of the Supporting Information for more information).
**Table S3G**. Additional model selection for LRS for parents in experiment C. Showing the top three models (with the zero‐inflation components from above) with additional dispersion parameters shown in order of AIC (see the starting of the Supporting Information for more information).
**Table S3H**. Summary table for the best identified LRS model for parents in experiment C.
**Table S3I**. Model selection for LRS for offspring in experiment A. Showing the top three models in order of AIC (see the starting of the Supporting Information for more information).
**Table S3H**. Additional model selection for LRS for offspring in experiment A. Showing the top two models (with the zero‐inflation components from above) with additional dispersion parameters shown in order of AIC (see the starting of the Supporting Information for more information).
**Table S3H**. Summary table for the best identified LRS model for offspring in experiment A.
**Table S3I**. Model selection for LRS for offspring in experiment B. Showing the top three models in order of AIC (see the starting of the Supporting Information for more information).
**Table S3J**. Summary table for the best identified LRS model for offspring in experiment B.
**Table S3K**. Model selection for LRS for parents in offspring in experiment C. Showing the top three models in order of AIC (see the starting of the Supporting Information for more information).
**Table S3L**. Summary table for the best identified LRS model for offspring in experiment C.
**Table S4A**. Full model output of individual fitness for parents in experiment A.
**Table S4B**. Full model output of individual fitness for parents in experiment B.
**Table S4C**. Full model output of individual fitness for parents in experiment C.
**Table S4D**. Full model output of individual fitness for offspring in experiment A.
**Table S4E**. Full model output of individual fitness for offspring in experiment B.
**Table S4F**. Full model output of individual fitness for offspring in experiment C.
**Table S5A**. Full model output of health span (speed) for parents in experiment A.
**Table S5B**. Full model output of health span (speed) for parents in experiment B.
**Table S5C**. Full model output of health span (speed) for parents in experiment C.
**Table S5D**. Full model output of health span (speed) for offspring in experiment A.
**Table S5E**. Full model output of health span (speed) for offspring in experiment B.
**Table S5F**. Full model output of health span (speed) for offspring in experiment C.
**Table S6A**. Full model output of health span (turns) for parents in experiment A.
**Table S6B**. Full model output of health span (turns) for parents in experiment B.
**Table S6C**. Full model output of health span (turns) for parents in experiment C.
**Table S6D**. Full model output of health span (turns) for offspring in experiment A.
**Table S6E**. Full model output of health span (turns) for offspring in experiment B.
**Table S6F**. Full model output of health span (turns) for offspring in experiment C.
**Table S7**. Primer sequences. Forward (fwd) and reverse (rev) sequences are listed in the 5’ to 3’ direction. Sequences acquired from [1] for *daf‐2* and [2] for the *actin‐3* reference gene.
**Table S8**. Relative gene expression (Δ*Ct*).
**Figure S1**. Age‐specific reproductive success (ARS) for parental and offspring generations across three experiments and four blocks (see Fig. 1 in the main MS legend for details)
**Figure S2**. Survival curves (with matricide) for parental and offspring generations across three experiments and four blocks (see Fig. 1 in the main MS legend for details).
**Figure S3**. Survival curves (without matricide) for parental and offspring generations across three experiments and four blocks (see Fig. 1 in the main MS legend for details).
**Figure S4**. Individual fitness for parental and offspring generations across all three experiments (see Fig. 1 in the main MS legend for details).
**Figure S4**. Normalized *daf‐2* expression following RNAi treatment versus untreated controls.
