# Supplementary material for: Ageing as “early‐life inertia”: Disentangling life‐history trade‐offs along a lifetime of an individual
Source: Evol Lett. 2021 Sep 8;5(5):551–64. doi: 10.1002/evl3.254 (PMC8484722; doi:10.1002/evl3.254)
Supplement: Supplementary file 1 — Table S1A. Full model output from the event history analysis excluding matricide for parents in experiment A. Table S1B. Full model output from the event history analysis including matricide for parents in experiment A. Table S1C. Full model output from the event history analysis excluding matricide for parents in experiment B. Table S1D. Full model output from the event history analysis including matricide for parents in experiment B. Table S1E. Full model output from the event history analysis excluding matricide for parents in experiment C. Table S1F. Full model output from the event history analysis including matricide for parents in experiment C. Table S1G. Full model output from the event history analysis excluding matricide for offspring in experiment A. Table S1H. Full model output from the event history analysis including matricide for offspring in experiment A. Table S1I. Full model output from the event history analysis excluding matricide for offspring in experiment B. Table S1J. Full model output from the event history analysis including matricide for offspring in experiment B. Table S1K. Full model output from the event history analysis excluding matricide for offspring in experiment C. Table S1L. Full model output from the event history analysis including matricide for offspring in experiment C. Table S2A. Model selection for ARS for parents in experiment A. Table S2B. Summary table for the best identified ARS model for parents in experiment A. Table S2C. Model selection for ARS for parents in experiment B. Showing the top three models in order of AIC (see the starting of the Supporting Information for more information). Table S2D. Additional model selection for ARS for parents in experiment B. Showing the top three models (with the zero‐inflation components from above) with additional dispersion parameters shown in order of AIC (see the starting of the Supporting Information for more information). Table S2E. Summary table for the best identified ARS [file EVL3-5-551-s001.docx]

**Supplementary Material:** Ageing as “early‐life inertia”: Disentangling life‐history trade‐offs along a lifetime of an individual

*Hanne Carlsson, Edward Ivimey-Cook, Elizabeth M. L. Duxbury, Nathan Edden, Kris Sales and Alexei A. Maklakov**

For ARS, LRS and Healthspan (Turns) model selection: Models with either too low or too high zero-inflation, dispersion, or uniformity parameters (identified using the DHARMa package) were omitted from selection. The model denoted with bold is the chosen “best” fitting model in terms of AIC and parsimony (the model with the lowest number of df within Δ6 AIC of the top model). In all cases the models have the same fixed and random effect structure which is omitted from this table (see Methods). In some cases, if the level of over- or under-dispersion was small (but still significant, denoted with a *), additional model selection was run to identify the most appropriate additional dispersion parameter incorporated into the “best” model. If the additional parameter resulted in significantly greater under-dispersion or if adding this parameter did not improve fit, the original model was instead selected.

**Table S1A.** Full model output from the event history analysis excluding matricide for parents in experiment A.

| *Predictors* | *Log-Odds* | *std. Error* | *Statistic* | *p* |
| --- | --- | --- | --- | --- |
| (Intercept) | -4.07 | 0.82 | -4.98 | **<0.001** |
| Treatment [ev] | 1.57 | 0.25 | 6.18 | **<0.001** |
| Block [2] | 1.65 | 0.27 | 6.09 | **<0.001** |
| Block [3] | 3.01 | 0.36 | 8.33 | **<0.001** |
| Block [4] | 3.64 | 0.40 | 9.03 | **<0.001** |
| *Random Effects* | | | | |
| σ^2^ | 3.29 | | | |
| τ_00_ _Set_ | 0.00 | | | |
| τ_00_ _ID_ | 0.00 | | | |
| τ_00_ _Day_ | 10.78 | | | |
| N _Set_ | 16 | | | |
| N _ID_ | 185 | | | |
| N _Day_ | 19 | | | |
| Observations | 1765 | | | |

**Table S1B.** Full model output from the event history analysis including matricide for parents in experiment A.

| *Predictors* | *Log-Odds* | *std. Error* | *Statistic* | *p* |
| --- | --- | --- | --- | --- |
| (Intercept) | -3.37 | 0.73 | -4.60 | **<0.001** |
| Treatment [ev] | 0.63 | 0.17 | 3.61 | **<0.001** |
| Block [2] | 1.67 | 0.32 | 5.28 | **<0.001** |
| Block [3] | 3.29 | 0.46 | 7.17 | **<0.001** |
| Block [4] | 4.26 | 0.54 | 7.88 | **<0.001** |
| *Random Effects* | | | | |
| σ^2^ | 3.29 | | | |
| τ_00_ _Set_ | 0.02 | | | |
| τ_00_ _ID_ | 0.20 | | | |
| τ_00_ _Day_ | 8.84 | | | |
| N _Set_ | 16 | | | |
| N _ID_ | 320 | | | |
| N _Day_ | 19 | | | |
| Observations | 2458 | | | |

**Table S1C.** Full model output from the event history analysis excluding matricide for parents in experiment B.

| *Predictors* | *Log-Odds* | *std. Error* | *Statistic* | *p* |
| --- | --- | --- | --- | --- |
| (Intercept) | -0.14 | 0.82 | -0.17 | 0.866 |
| Treatment [ev] | 1.75 | 0.25 | 6.94 | **<0.001** |
| Block [2] | -2.78 | 0.35 | -8.05 | **<0.001** |
| Block [3] | -3.20 | 0.36 | -8.97 | **<0.001** |
| Block [4] | -1.55 | 0.36 | -4.27 | **<0.001** |
| *Random Effects* | | | | |
| σ^2^ | 3.29 | | | |
| τ_00_ _Set_ | 0.00 | | | |
| τ_00_ _ID_ | 0.00 | | | |
| τ_00_ _Day_ | 9.22 | | | |
| N _Set_ | 17 | | | |
| N _ID_ | 179 | | | |
| N _Day_ | 16 | | | |
| Observations | 1471 | | | |

**Table S1D.** Full model output from the event history analysis including matricide for parents in experiment B.

| *Predictors* | *Log-Odds* | *std. Error* | *Statistic* | *p* |
| --- | --- | --- | --- | --- |
| (Intercept) | 0.05 | 0.66 | 0.07 | 0.945 |
| Treatment [ev] | 0.95 | 0.15 | 6.19 | **<0.001** |
| Block [2] | -2.22 | 0.25 | -8.73 | **<0.001** |
| Block [3] | -2.58 | 0.27 | -9.72 | **<0.001** |
| Block [4] | -0.88 | 0.24 | -3.73 | **<0.001** |
| *Random Effects* | | | | |
| σ^2^ | 3.29 | | | |
| τ_00_ _Set2_ | 0.02 | | | |
| τ_00_ _ID_ | 0.00 | | | |
| τ_00_ _Day_ | 6.01 | | | |
| N _Set_ | 17 | | | |
| N _ID_ | 319 | | | |
| N _Day_ | 16 | | | |
| Observations | 2219 | | | |

**Table S1E.** Full model output from the event history analysis excluding matricide for parents in experiment C.

| *Predictors* | *Log-Odds* | *std. Error* | *Statistic* | *p* |
| --- | --- | --- | --- | --- |
| (Intercept) | -5.54 | 1.43 | -3.87 | **<0.001** |
| Treatment [ev] | 3.09 | 0.25 | 12.17 | **<0.001** |
| Block [2] | -0.76 | 0.25 | -3.04 | **0.002** |
| Block [3] | 1.84 | 0.26 | 7.19 | **<0.001** |
| Block [4] | 2.60 | 0.28 | 9.34 | **<0.001** |
| *Random Effects* | | | | |
| σ^2^ | 3.29 | | | |
| τ_00_ _Set_ | 0.00 | | | |
| τ_00_ _ID_ | 0.00 | | | |
| τ_00_ _Day_ | 29.44 | | | |
| N _Set_ | 16 | | | |
| N _ID_ | 291 | | | |
| N _Day_ | 17 | | | |
| Observations | 3211 | | | |

**Table S1F.** Full model output from the event history analysis including matricide for parents in experiment C.

| *Predictors* | *Log-Odds* | *std. Error* | *Statistic* | *p* |
| --- | --- | --- | --- | --- |
| Intercept) | -4.14 | 1.02 | -4.07 | **<0.001** |
| Treatment [ev] | 2.08 | 0.19 | 10.73 | **<0.001** |
| Block [2] | -0.21 | 0.22 | -0.98 | 0.327 |
| Block [3] | 1.48 | 0.22 | 6.62 | **<0.001** |
| Block [4] | 2.02 | 0.24 | 8.59 | **<0.001** |
| *Random Effects* | | | | |
| σ^2^ | 3.29 | | | |
| τ_00_ _Se_ | 0.00 | | | |
| τ_00_ _ID_ | 0.00 | | | |
| τ_00_ _Day_ | 15.83 | | | |
| N _Set_ | 16 | | | |
| N _ID_ | 319 | | | |
| N _Day_ | 17 | | | |
| Observations | 3412 | | | |

**Table S1G.** Full model output from the event history analysis excluding matricide for offspring in experiment A.

| *Predictors* | *Log-Odds* | *std. Error* | *Statistic* | *p* |
| --- | --- | --- | --- | --- |
| (Intercept) | -1.88 | 0.74 | -2.56 | **0.011** |
| Treatment [ev] | -0.10 | 0.20 | -0.51 | 0.611 |
| Block [2] | -0.09 | 0.25 | -0.35 | 0.728 |
| Block [3] | 0.70 | 0.26 | 2.72 | **0.007** |
| Block [4] | 0.35 | 0.40 | 0.89 | 0.373 |
| *Random Effects* | | | | |
| σ^2^ | 3.29 | | | |
| τ_00_ _Set_ | 0.00 | | | |
| τ_00_ _ID_ | 0.00 | | | |
| τ_00_ _Day_ | 5.57 | | | |
| N _Set_ | 16 | | | |
| N _ID_ | 177 | | | |
| N _Day_ | 12 | | | |
| Observations | 1290 | | | |

**Table S1H.** Full model output from the event history analysis including matricide for offspring in experiment A.

| *Predictors* | *Log-Odds* | *std. Error* | *Statistic* | *p* |
| --- | --- | --- | --- | --- |
| (Intercept) | -1.73 | 0.74 | -2.34 | **0.019** |
| Treatment [ev] | -0.07 | 0.17 | -0.41 | 0.683 |
| Block [2] | 0.06 | 0.20 | 0.32 | 0.750 |
| Block [3] | 0.41 | 0.24 | 1.76 | 0.078 |
| Block [4] | 0.17 | 0.33 | 0.53 | 0.598 |
| *Random Effects* | | | | |
| σ^2^ | 3.29 | | | |
| τ_00_ _Set_ | 0.00 | | | |
| τ_00_ _ID_ | 0.00 | | | |
| τ_00_ _Day_ | 5.73 | | | |
| N _Set_ | 16 | | | |
| N _ID_ | 225 | | | |
| N _Day_ | 12 | | | |
| Observations | 1594 | | | |

**Table S1I.** Full model output from the event history analysis excluding matricide for offspring in experiment B.

| *Predictors* | *Log-Odds* | *std. Error* | *Statistic* | *p* |
| --- | --- | --- | --- | --- |
| (Intercept) | -1.41 | 0.55 | -2.54 | **0.011** |
| Treatment [ev] | -0.05 | 0.20 | -0.27 | 0.789 |
| Block [2] | 0.52 | 0.28 | 1.90 | 0.058 |
| Block [3] | -0.25 | 0.27 | -0.93 | 0.355 |
| Block [4] | -0.13 | 0.29 | -0.44 | 0.660 |
| *Random Effects* | | | | |
| σ^2^ | 3.29 | | | |
| τ_00_ _Set_ | 0.00 | | | |
| τ_00 ID_ | 0.00 | | | |
| τ_00_ _Day_ | 2.98 | | | |
| N _Set_ | 16 | | | |
| N _ID_ | 159 | | | |
| N _Day_ | 13 | | | |
| Observations | 1062 | | | |

**Table S1J.** Full model output from the event history analysis including matricide for offspring in experiment B.

| *Predictors* | *Log-Odds* | *std. Error* | *Statistic* | *p* |
| --- | --- | --- | --- | --- |
| (Intercept) | -1.42 | 0.61 | -2.35 | **0.019** |
| Treatment [ev] | -0.10 | 0.16 | -0.65 | 0.518 |
| Block [2] | 0.77 | 0.26 | 2.99 | **0.003** |
| Block [3] | 0.09 | 0.24 | 0.35 | 0.728 |
| Block [4] | -0.09 | 0.28 | -0.33 | 0.741 |
| *Random Effects* | | | | |
| σ^2^ | 3.29 | | | |
| τ_00_ _Set_ | 0.02 | | | |
| τ_00_ _ID_ | 0.00 | | | |
| τ_00_ _Day_ | 3.77 | | | |
| N _Se_ | 16 | | | |
| N _ID_ | 241 | | | |
| N _Day_ | 13 | | | |
| Observations | 1535 | | | |

**Table S1K.** Full model output from the event history analysis excluding matricide for offspring in experiment C.

| Predictors | Log-Odds | std. Error | Statistic | p |
| --- | --- | --- | --- | --- |
| (Intercept) | -2.07 | 1.22 | -1.70 | 0.090 |
| Treatment [ev] | -0.17 | 0.19 | -0.87 | 0.382 |
| Block [2] | -2.06 | 0.42 | -4.89 | **<0.001** |
| Block [3] | -0.28 | 0.31 | -0.89 | 0.374 |
| Block [4] | 0.40 | 0.34 | 1.17 | 0.240 |
| *Random Effects* | | | | |
| σ^2^ | 3.29 | | | |
| τ_00_ _Set_ | 0.02 | | | |
| τ_00_ _ID_ | 0.16 | | | |
| τ_00_ _Day_ | 17.45 | | | |
| N _Set_ | 16 | | | |
| N _ID_ | 238 | | | |
| N _Day_ | 14 | | | |
| Observations | 2304 | | | |

**Table S1L.** Full model output from the event history analysis including matricide for offspring in experiment C.

| Predictors | Log-Odds | std. Error | Statistic | p |
| --- | --- | --- | --- | --- |
| (Intercept) | -1.66 | 0.96 | -1.72 | 0.085 |
| Treatment [ev] | -0.10 | 0.15 | -0.70 | 0.485 |
| Block [2] | -1.60 | 0.31 | -5.11 | **<0.001** |
| Block [3] | -0.33 | 0.22 | -1.50 | 0.133 |
| Block [4] | 0.29 | 0.21 | 1.35 | 0.177 |
| *Random Effects* | | | | |
| σ^2^ | 3.29 | | | |
| τ_00_ _Set_ | 0.00 | | | |
| τ_00 ID_ | 0.02 | | | |
| τ_00_ _Day_ | 11.46 | | | |
| N _Set_ | 16 | | | |
| N _ID_ | 311 | | | |
| N _Day_ | 14 | | | |
| Observations | 2837 | | | |

**Table S2A.** Model selection for ARS for parents in experiment A. Showing the top three models in order of AIC (see start of supplementary/methods for more information).

| *Family* | *ziFormula* | *zi* | *di* | *AIC* | *df* |
| --- | --- | --- | --- | --- | --- |
| genpois | **~Treatment * Day + Day2 + Block** | **1.002** | **0.846*** | **10816.316** | **20** |
| genpois | ~Block + Day | 1.035 | 0.831 | 10836.247 | 17 |
| genpois | ~Treatment * Day + Treatment * Day2 | 0.990 | 0.889 | 11079.373 | 18 |

**Table S2B.** Summary table for the best identified ARS model for parents in experiment A.

| *Predictors* | *Estimates* | *std. Error* | *Statistic* | *p* |
| --- | --- | --- | --- | --- |
| (Intercept) | 2.61 | 0.15 | 17.39 | **<0.001** |
| Treatment [ev] | -1.46 | 0.21 | -6.84 | **<0.001** |
| Day | 0.85 | 0.10 | 8.64 | **<0.001** |
| Day2 | -0.17 | 0.02 | -10.37 | **<0.001** |
| Block [2] | -0.38 | 0.08 | -4.68 | **<0.001** |
| Block [3] | -0.69 | 0.08 | -8.33 | **<0.001** |
| Block [4] | -0.75 | 0.08 | -9.00 | **<0.001** |
| Treatment [ev] * Day | 1.49 | 0.16 | 9.53 | **<0.001** |
| Treatment [ev] * Day2 | -0.28 | 0.03 | -10.44 | **<0.001** |
| *Zero-Inflated Model* | | | | |
| (Intercept) | 11.03 | 1.18 | 9.37 | **<0.001** |
| Treatment [ev] | -2.54 | 0.64 | -3.98 | **<0.001** |
| Day | -9.22 | 0.91 | -10.10 | **<0.001** |
| Day2 | 1.23 | 0.14 | 8.56 | **<0.001** |
| Block [2] | -3.10 | 0.53 | -5.89 | **<0.001** |
| Block [3] | -16.39 | 114.60 | -0.14 | 0.886 |
| Block [4] | -16.94 | 148.45 | -0.11 | 0.909 |
| Treatment [ev] * Day | 1.39 | 0.33 | 4.17 | **<0.001** |
| *Random Effects* | | | | |
| σ^2^ | 0.48 | | | |
| τ_00_ _Set_ | 0.00 | | | |
| τ_00_ _ID_ | 0.17 | | | |
| N _Set_ | 16 | | | |
| N _ID_ | 320 | | | |
| Observations | 1491 | | | |

**Table S2C.** Model selection for ARS for parents in experiment B. Showing the top three models in order of AIC (see start of supplementary/methods for more information).

| *Family* | *ziFormula* | *zi* | *di* | *AIC* | *df* |
| --- | --- | --- | --- | --- | --- |
| genpois | ~Treatment + Block | 0.863 | 0.813 | 10994.7123 | 17 |
| genpois | **~Block** | **0.859** | **0.822*** | **10999.9153** | **16** |
| genpois | ~Treatment + Day | 0.878 | 0.809 | 11018.2176 | 15 |

**Table S2D.** Additional model selection for ARS for parents in experiment B. Showing the top three models (with the zero-inflation components from above) with additional dispersion parameters shown in order of AIC (see start of supplementary/methods for more information).

| *dispFormula* | *zi* | *di* | *AIC* | *df* |
| --- | --- | --- | --- | --- |
| ~ Treatment * Day + Treatment * Day2 | 0.924 | 0.801 | 10944.759 | 21 |
| ~ Treatment * Day + Day2 | **0.922** | **0.812*** | **10946.314** | **20** |
| ~ Treatment * Day2 + Day | 0.923 | 0.814 | 10949.474 | 20 |

**Table S2E.** Summary table for the best identified ARS model for parents in experiment B.

| *Predictors* | *Log-Mean* | *std. Error* | *Statistic* | *p* |
| --- | --- | --- | --- | --- |
| (Intercept) | -0.09 | 0.16 | -0.53 | 0.595 |
| Treatment [ev] | 0.11 | 0.21 | 0.52 | 0.604 |
| Day | 2.25 | 0.10 | 22.65 | **<0.001** |
| Day2 | -0.43 | 0.02 | -26.60 | **<0.001** |
| Block [2] | 1.03 | 0.09 | 11.64 | **<0.001** |
| Block [3] | 1.19 | 0.09 | 13.44 | **<0.001** |
| Block [4] | 0.59 | 0.09 | 6.78 | **<0.001** |
| Treatment [ev] * Day | -0.07 | 0.15 | -0.48 | 0.635 |
| Treatment [ev] * Day2 | 0.00 | 0.03 | 0.01 | 0.992 |
| *Zero-Inflated Model* |  |  |  |  |
| (Intercept) | -3.51 | 0.57 | -6.14 | **<0.001** |
| Block [2] | 2.03 | 0.59 | 3.46 | **0.001** |
| Block [3] | 0.07 | 0.66 | 0.11 | 0.911 |
| Block [4] | -0.11 | 0.65 | -0.17 | 0.867 |
| *Dispersion Model* | | | | |
| (Intercept) | 2.05 | 0.36 | 5.65 | **<0.001** |
| Treatment [ev] | -0.41 | 0.29 | -1.45 | 0.147 |
| Day | 0.27 | 0.27 | 0.98 | 0.327 |
| Day2 | -0.10 | 0.04 | -2.22 | **0.027** |
| Treatment [ev] * Day | 0.35 | 0.09 | 3.65 | **<0.001** |
| *Random Effects* | | | | |
| σ^2^ | 0.25 | | | |
| τ_00_ _Set_ | 0.00 | | | |
| τ_00_ _ID_ | 0.14 | | | |
| N _Set_ | 16 | | | |
| N _ID_ | 319 | | | |
| Observations | 1488 | | | |

**Table S2F.** Model selection for ARS for parents in experiment C. Showing the top three models in order of AIC (see start of supplementary/methods for more information).

| *Family* | *ziFormula* | *zi* | *di* | *AIC* | *df* |
| --- | --- | --- | --- | --- | --- |
| genpois | **~ Treatment * Day2 + Day + Block** | **0.906** | **0.818*** | **10473.444** | **20** |
| genpois | ~ Treatment + Day + Day2 | 0.910 | 0.818 | 10482.209 | 16 |
| genpois | ~ Block | 0.908 | 0.818 | 10486.808 | 16 |

**Table S2G.** Additional model selection for ARS for parents in experiment C. Showing the top two models (with the zero-inflation components from above) with additional dispersion parameters shown in order of AIC (see start of supplementary/methods for more information).

| *dispFormula* | *zi* | *di* | *AIC* | *df* |
| --- | --- | --- | --- | --- |
| ~ Treatment | **0.907** | **0.816** | **10466.325** | **21** |
| ~ 1 | 0.906 | 0.818 | 10473.444 | 20 |

**Table S2H.** Summary table for the best identified ARS model for parents in experiment C.

| *Predictors* | *Log-Mean* | *std. Error* | *Statistic* | *p* |
| --- | --- | --- | --- | --- |
| (Intercept) | 2.43 | 0.10 | 24.52 | **<0.001** |
| Treatment [ev] | 0.18 | 0.16 | 1.16 | 0.247 |
| Day | 2.86 | 0.10 | 28.00 | **<0.001** |
| Day2 | -0.80 | 0.02 | -32.92 | **<0.001** |
| Block [2] | -0.49 | 0.04 | -12.70 | **<0.001** |
| Block [3] | -0.18 | 0.04 | -4.95 | **<0.001** |
| Block [4] | -0.31 | 0.04 | -8.22 | **<0.001** |
| Treatment [ev] * Day | -0.13 | 0.17 | -0.74 | 0.459 |
| Treatment [ev] * Day2 | 0.01 | 0.04 | 0.24 | 0.811 |
| *Zero-Inflated Model* | | | | |
| (Intercept) | -29.99 | 2403.06 | -0.01 | 0.990 |
| Treatment [ev] | -6.52 | 4.23 | -1.54 | 0.123 |
| Day2 | -2.51 | 1.16 | -2.16 | **0.031** |
| Day | 9.79 | 4.24 | 2.31 | **0.021** |
| Block [2] | 15.22 | 2403.06 | 0.01 | 0.995 |
| Block [3] | 15.04 | 2403.06 | 0.01 | 0.995 |
| Block [4] | 17.07 | 2403.06 | 0.01 | 0.994 |
| Treatment [ev] * Day2 | 1.22 | 0.70 | 1.74 | 0.083 |
| *Dispersion Model* | | | | |
| (Intercept) | 2.23 | 0.08 | 28.39 | **<0.001** |
| Treatment [ev] | 0.35 | 0.12 | 3.01 | **0.003** |
| *Random Effects* | | | | |
| σ^2^ | 0.11 | | | |
| τ_00_ _Set_ | 0.00 | | | |
| τ_00_ _ID_ | 0.00 | | | |
| N _Set_ | 16 | | | |
| N _ID_ | 320 | | | |
| Observations | 1573 | | | |

**Table S2I.** Model selection for ARS for offspring in experiment A. Showing the top three models in order of AIC (see start of supplementary/methods for more information).

| *Family* | *ziFormula* | *zi* | *di* | *AIC* | *df* |
| --- | --- | --- | --- | --- | --- |
| genpois | ~ Treatment * Day + Treatment * Day2 + Block | 1.014 | 0.615 | 5407.702 | 21 |
| genpois | **~ Block + Day** | **0.991** | **0.748*** | **5410.548** | **17** |
| genpois | ~ Treatment + Day + Day2 + Block | 0.997 | 0.714 | 5411.181 | 19 |

**Table S2J.** Summary table for the best identified ARS model for offspring in experiment A.

| *Predictors* | *Log-Mean* | *std. Error* | *Statistic* | *p* |
| --- | --- | --- | --- | --- |
| (Intercept) | 0.81 | 0.29 | 2.82 | **0.005** |
| Treatment [ev] | -0.56 | 0.44 | -1.28 | 0.201 |
| Day | 2.59 | 0.19 | 13.57 | **<0.001** |
| Day2 | -0.47 | 0.03 | -15.00 | **<0.001** |
| Block [2] | -0.57 | 0.11 | -4.96 | **<0.001** |
| Block [3] | -1.18 | 0.43 | -2.73 | **0.006** |
| Block [4] | -1.02 | 0.36 | -2.86 | **0.004** |
| Treatment [ev] * Day | 0.34 | 0.30 | 1.16 | 0.244 |
| Treatment [ev] * Day2 | -0.07 | 0.05 | -1.44 | 0.150 |
| *Zero-Inflated Model* | | | | |
| (Intercept) | 1.11 | 0.30 | 3.74 | **<0.001** |
| Block [2] | 1.88 | 0.26 | 7.28 | **<0.001** |
| Block [3] | 7.35 | 0.57 | 12.99 | **<0.001** |
| Block [4] | 6.22 | 0.57 | 10.84 | **<0.001** |
| Day | -1.61 | 0.14 | -11.72 | **<0.001** |
| *Random Effects* | | | | |
| σ^2^ | 0.58 | | | |
| τ_00_ _Set_ | 0.01 | | | |
| τ_00_ _ID_ | 0.10 | | | |
| N _Set_ | 16 | | | |
| N _ID_ | 215 | | | |
| Observations | 1009 | | | |

**Table S2K.** Model selection for ARS for offspring in experiment B. Showing the top three models in order of AIC (see start of supplementary/methods for more information).

| *Family* | *ziFormula* | *zi* | *di* | *AIC* | *df* |
| --- | --- | --- | --- | --- | --- |
| genpois | **~Treatment * Day + Day2 + Block** | **0.991** | **0.260*** | **4745.673** | **20** |
| genpois | ~Treatment * Day2 + Day + Block | 0.991 | 0.259 | 4745.970 | 20 |
| genpois | ~Treatment * Day + Treatment * Day2 + Block | 0.991 | 0.261 | 4747.602 | 21 |

**Table S2L.** Summary table for the best identified ARS model for offspring in experiment B.

| *Predictors* | *Log-Mean* | *std. Error* | *Statistic* | *p* |
| --- | --- | --- | --- | --- |
| (Intercept) | -3.44 | 0.64 | -5.35 | **<0.001** |
| Treatment [ev] | -0.46 | 0.63 | -0.72 | 0.469 |
| Day | 2.22 | 0.25 | 8.93 | **<0.001** |
| Day2 | -0.34 | 0.04 | -9.40 | **<0.001** |
| Block [2] | 2.28 | 0.59 | 3.89 | **<0.001** |
| Block [3] | 2.95 | 0.59 | 5.05 | **<0.001** |
| Block [4] | 0.51 | 0.66 | 0.78 | 0.436 |
| Treatment [ev] * Day | 0.33 | 0.36 | 0.92 | 0.355 |
| Treatment [ev] * Day2 | -0.05 | 0.05 | -0.91 | 0.364 |
| *Zero-Inflated Model* | | | | |
| (Intercept) | 7.15 | 1.16 | 6.18 | **<0.001** |
| Treatment [ev] | -0.48 | 0.88 | -0.54 | 0.591 |
| Day | -3.19 | 0.76 | -4.19 | **<0.001** |
| Day2 | 0.07 | 0.18 | 0.38 | 0.707 |
| Block [2] | -2.72 | 0.76 | -3.58 | **<0.001** |
| Block [3] | -2.65 | 0.74 | -3.57 | **<0.001** |
| Block [4] | 2.33 | 0.95 | 2.45 | **0.014** |
| Treatment [ev] * Day | 0.58 | 0.45 | 1.28 | 0.199 |
| *Random Effects* | | | | |
| σ^2^ | 0.00 | | | |
| τ_00_ _Set_ | 0.39 | | | |
| τ_00_ _ID_ | 1.69 | | | |
| N _Set_ | 16 | | | |
| N _ID_ | 234 | | | |
| Observations | 1077 | | | |

**Table S2M.** Model selection for ARS for offspring in experiment C. Showing the top three models in order of AIC (see start of supplementary/methods for more information).

| *Family* | *ziFormula* | *zi* | *di* | *AIC* | *df* |
| --- | --- | --- | --- | --- | --- |
| genpois | **~Day + Day2** | **0.995** | **0.804** | **11475.736** | **15** |
| genpois | ~Treatment + Day + Day2 | 0.995 | 0.804 | 11477.736 | 16 |
| genpois | ~Treatment * Day + Day2 | 0.996 | 0.804 | 11478.624 | 17 |

**Table S2N.** Summary table for the best identified ARS model for offspring in experiment C.

| *Predictors* | *Log-Mean* | *std. Error* | *Statistic* | *p* |
| --- | --- | --- | --- | --- |
| (Intercept) | 2.86 | 0.16 | 17.96 | **<0.001** |
| Treatment [ev] | -0.41 | 0.22 | -1.91 | 0.056 |
| Day | 1.48 | 0.13 | 11.68 | **<0.001** |
| Day2 | -0.35 | 0.02 | -14.24 | **<0.001** |
| Block [2] | -0.02 | 0.06 | -0.33 | 0.741 |
| Block [3] | -0.45 | 0.07 | -6.75 | **<0.001** |
| Block [4] | -0.59 | 0.06 | -9.15 | **<0.001** |
| Treatment [ev] * Day | 0.19 | 0.17 | 1.11 | 0.265 |
| Treatment [ev] * Day2 | -0.02 | 0.03 | -0.74 | 0.462 |
| *Zero-Inflated Model* | | | | |
| (Intercept) | 1.00 | 0.34 | 2.92 | **0.003** |
| Day | -2.50 | 0.31 | -8.12 | **<0.001** |
| Day2 | 0.43 | 0.05 | 8.11 | **<0.001** |
| *Random Effects* | | | | |
| σ^2^ | 0.55 | | | |
| τ_00_ _Set_ | 0.00 | | | |
| τ_00_ _ID_ | 0.03 | | | |
| N _Set_ | 16 | | | |
| N _ID_ | 311 | | | |
| Observations | 1531 | | | |

**Table S3A.** Model selection for LRS for parents in experiment A. Showing the top three models in order of AIC (see start of supplementary/methods for more information).

| *Family* | *ziFormula* | *zi* | *di* | *AIC* | *df* |
| --- | --- | --- | --- | --- | --- |
| compois | **~1** | **1.009** | **0.916** | **3160.763** | **8** |
| compois | ~Block | 1.020 | 0.915 | 3162.304 | 11 |
| compois | ~Treatment | 1.008 | 0.920 | 3162.456 | 9 |

**Table S3B.** Summary table for the best identified LRS model for parents in experiment A.

| *Predictors* | *Estimates* | *std. Error* | *Statistic* | *p* |
| --- | --- | --- | --- | --- |
| (Intercept) | 4.79 | 0.06 | 82.56 | **<0.001** |
| Treatment [ev] | 0.29 | 0.05 | 5.72 | **<0.001** |
| Block [2] | -0.21 | 0.08 | -2.73 | **0.006** |
| Block [3] | -0.55 | 0.08 | -6.98 | **<0.001** |
| Block [4] | -0.62 | 0.08 | -7.47 | **<0.001** |
| *Zero-Inflated Model* | | | | |
| (Intercept) | -4.66 | 0.61 | -7.63 | **<0.001** |
| *Random Effects* | | | | |
| τ_00_ _Set_ | 0.00 | | | |
| N _Set_ | 16 | | | |
| Observations | 303 | | | |

**Table S3C.** Model selection for LRS for parents in experiment B. Showing the top three models in order of AIC (see start of supplementary/methods for more information).

| *Family* | *ziFormula* | *zi* | *di* | *AIC* | *df* |
| --- | --- | --- | --- | --- | --- |
| compois | **~1** | **0.762** | **0.907*** | **3285.019** | **8** |
| compois | ~Treatment | 0.812 | 0.905 | 3285.634 | 9 |
| compois | ~Block | 0.755 | 0.900 | 3288.269 | 11 |

**Table S3D.** Additional model selection for LRS for parents in experiment B. Showing the top three models (with the zero-inflation components from above) with additional dispersion parameters shown in order of AIC (see start of supplementary/methods for more information).

| *dispFormula* | *zi* | *di* | *AIC* | *df* |
| --- | --- | --- | --- | --- |
| ~ Block + Treatment | **0.511** | **0.961** | **3231.345** | **12** |
| ~ Block | 0.521 | 0.980 | 3243.927 | 11 |
| ~ Treatment | 0.720 | 0.907 | 3277.189 | 9 |

**Table S3E.** Summary table for the best identified LRS model for parents in experiment B.

| *Predictors* | *Estimates* | *std. Error* | *Statistic* | *p* |
| --- | --- | --- | --- | --- |
| (Intercept) | 3.91 | 0.08 | 47.86 | **<0.001** |
| Treatment [ev] | -0.05 | 0.04 | -1.11 | 0.269 |
| Block [2] | 0.92 | 0.09 | 10.55 | **<0.001** |
| Block [3] | 1.15 | 0.08 | 13.59 | **<0.001** |
| Block [4] | 0.61 | 0.11 | 5.48 | **<0.001** |
| *Zero-Inflated Model* | | | | |
| (Intercept) | -5.75 | 1.00 | -5.74 | **<0.001** |
| *Dispersion Model* | | | | |
| (Intercept) | 3.46 | 0.34 | 10.26 | **<0.001** |
| Block [2] | -1.24 | 0.37 | -3.34 | **0.001** |
| Block [3] | -1.54 | 0.37 | -4.17 | **<0.001** |
| Block [4] | 0.23 | 0.43 | 0.53 | 0.599 |
| Treatment [ev] | 0.82 | 0.22 | 3.79 | **<0.001** |
| *Random Effects* | | | | |
| τ_00_ _Set_ | 0.00 | | | |
| N _Set_ | 16 | | | |
| Observations | 316 | | | |

**Table S3F.** Model selection for LRS for parents in experiment C. Showing the top three models in order of AIC (see start of supplementary/methods for more information).

| *Family* | *ziFormula* | *zi* | *di* | *AIC* | *df* |
| --- | --- | --- | --- | --- | --- |
| compois | ~0 | **NA** | **0.909*** | **3188.598** | **7** |
| poisson | ~0 | NA | 0.790 | 3304.392 | 7 |
| poisson | ~0 | NA | 2.015 | 4209.674 | 6 |

**Table S3G.** Additional model selection for LRS for parents in experiment C. Showing the top three models (with the zero-inflation components from above) with additional dispersion parameters shown in order of AIC (see start of supplementary/methods for more information).

| *dispFormula* | *zi* | *di* | *AIC* | *df* |
| --- | --- | --- | --- | --- |
| ~Block | **NA** | **0.941** | **3161.166** | **10** |
| ~Block + Treatment | NA | 0.940 | 3161.940 | 11 |
| ~1 | NA | 0.909 | 3188.598 | 7 |

**Table S3H.** Summary table for the best identified LRS model for parents in experiment C.

| *Predictors* | *Estimates* | *std. Error* | *Statistic* | *p* |
| --- | --- | --- | --- | --- |
| (Intercept) | 5.60 | 0.01 | 395.15 | **<0.001** |
| Treatment [ev] | -0.01 | 0.02 | -0.34 | 0.734 |
| Block [2] | -0.54 | 0.03 | -18.63 | **<0.001** |
| Block [3] | -0.19 | 0.02 | -7.93 | **<0.001** |
| Block [4] | -0.23 | 0.02 | -9.60 | **<0.001** |
| *Dispersion Model* | | | | |
| (Intercept) | 0.85 | 0.16 | 5.25 | **<0.001** |
| Block [2] | 1.29 | 0.23 | 5.57 | **<0.001** |
| Block [3] | 1.10 | 0.23 | 4.87 | **<0.001** |
| Block [4] | 1.15 | 0.23 | 5.06 | **<0.001** |
| *Random Effects* | | | | |
| τ_00_ _Set_ | 0.00 | | | |
| N _Set_ | 16 | | | |
| Observations | 317 | | | |

**Table S3I.** Model selection for LRS for offspring in experiment A. Showing the top three models in order of AIC (see start of supplementary/methods for more information).

| *Family* | *ziFormula* | *zi* | *di* | *AIC* | *df* |
| --- | --- | --- | --- | --- | --- |
| compois | **~Block** | **1.001** | **0.819*** | **1823.762** | **11** |
| compois | ~Treatment + Block | 1.007 | 0.826 | 1825.137 | 12 |
| poisson | ~1 | 1.013 | 0.929 | 5975.639 | 7 |

**Table S3H.** Additional model selection for LRS for offspring in experiment A. Showing the top two models (with the zero-inflation components from above) with additional dispersion parameters shown in order of AIC (see start of supplementary/methods for more information).

| *dispFormula* | *zi* | *di* | *AIC* | *df* |
| --- | --- | --- | --- | --- |
| ~Block + Treatment | **1.004** | **0.871** | **1804.107** | **15** |
| ~Treatment | 1.004 | 0.829 | 1822.832 | 12 |

**Table S3H.** Summary table for the best identified LRS model for offspring in experiment A.

| *Predictors* | *Estimates* | *std. Error* | *Statistic* | *p* |
| --- | --- | --- | --- | --- |
| (Intercept) | 5.25 | 0.05 | 103.86 | **<0.001** |
| Treatment [ev] | -0.13 | 0.07 | -1.83 | 0.068 |
| Block [2] | -0.62 | 0.10 | -6.43 | **<0.001** |
| Block [3] | -2.34 | 0.27 | -8.59 | **<0.001** |
| Block [4] | -2.51 | 0.33 | -7.63 | **<0.001** |
| *Zero-Inflated Model* | | | | |
| Intercept) | -4.25 | 1.01 | -4.22 | **<0.001** |
| Block [2] | 0.48 | 1.32 | 0.37 | 0.715 |
| Block [3] | 5.94 | 1.09 | 5.46 | **<0.001** |
| Block [4] | 3.99 | 1.12 | 3.55 | **<0.001** |
| *Dispersion Model* | | | | |
| (Intercept) | 2.90 | 0.24 | 12.32 | **<0.001** |
| Block [2] | 1.75 | 0.47 | 3.69 | **<0.001** |
| Block [3] | -0.26 | 1.14 | -0.22 | 0.822 |
| Block [4] | 15.50 | 3264.76 | 0.00 | 0.996 |
| *Random Effects* | | | | |
| τ_00_ _Set_ | 0.00 | | | |
| N _Set_ | 16 | | | |
| Observations | 210 | | | |

**Table S3I.** Model selection for LRS for offspring in experiment B. Showing the top three models in order of AIC (see start of supplementary/methods for more information).

| *Family* | *ziFormula* | *zi* | *di* | *AIC* | *df* |
| --- | --- | --- | --- | --- | --- |
| compois | **~Block** | **0.994** | **0.895** | **1947.021** | **11** |
| compois | ~Treatment + Block | 1.010 | 0.889 | 1948.723 | 12 |
| compois | ~1 | 1.029 | 0.895 | 2001.865 | 8 |

**Table S3J.** Summary table for the best identified LRS model for offspring in experiment B.

| *Predictors* | *Estimates* | *std. Error* | *Statistic* | *p* |
| --- | --- | --- | --- | --- |
| (Intercept) | 3.84 | 0.24 | 16.14 | **<0.001** |
| Treatment [ev] | -0.18 | 0.13 | -1.37 | 0.171 |
| Block [2] | 0.39 | 0.25 | 1.57 | 0.117 |
| Block [3] | 0.93 | 0.25 | 3.76 | **<0.001** |
| Block [4] | -0.49 | 0.31 | -1.61 | 0.10 |
| *Zero-Inflated Model* | | | | |
| (Intercept) | 0.56 | 0.33 | 1.72 | 0.085 |
| Block [2] | -2.56 | 0.50 | -5.08 | **<0.001** |
| Block [3] | -3.24 | 0.59 | -5.52 | **<0.001** |
| Block [4] | -0.62 | 0.46 | -1.37 | 0.171 |
| *Random Effects* | | | | |
| τ_00_ _Set_ | 0.00 | | | |
| N _Set_ | 16 | | | |
| Observations | 228 | | | |

**Table S3K.** Model selection for LRS for parents in offspring in experiment C. Showing the top three models in order of AIC (see start of supplementary/methods for more information).

| *Family* | *ziFormula* | *zi* | *di* | *AIC* | *df* |
| --- | --- | --- | --- | --- | --- |
| compois | **~1** | **1.040** | **0.931** | **3344.470** | **8** |
| compois | ~Treatment | 1.040 | 0.931 | 3346.470 | 9 |
| compois | ~Block | 0.949 | 0.924 | 3347.612 | 11 |

**Table S3L.** Summary table for the best identified LRS model for offspring in experiment C.

| *Predictors* | *Estimates* | *std. Error* | *Statistic* | *p* |
| --- | --- | --- | --- | --- |
| (Intercept) | 5.13 | 0.05 | 112.60 | **<0.001** |
| Treatment [ev] | -0.11 | 0.04 | -2.48 | **0.013** |
| Block [2] | 0.26 | 0.06 | 4.69 | **<0.001** |
| Block [3] | -0.15 | 0.06 | -2.50 | **0.012** |
| Block [4] | -0.53 | 0.07 | -8.03 | **<0.001** |
| *Zero-Inflated Model* | | | | |
| (Intercept) | -5.03 | 0.71 | -7.07 | **<0.001** |
| *Random Effects* | | | | |
| τ_00_ _Set_ | 0.00 | | | |
| N _Set_ | 16 | | | |
| Observations | 308 | | | |

**Table S4A.** Full model output of individual fitness for parents in experiment A.

| *Predictors* | *Estimates* | *std. Error* | *Statistic* | *p* |
| --- | --- | --- | --- | --- |
| (Intercept) | 2.60 | 0.07 | 39.93 | **<0.001** |
| Treatment [ev] | 0.25 | 0.06 | 4.43 | **<0.001** |
| Block [2] | 0.10 | 0.08 | 1.16 | 0.248 |
| Block [3] | 0.09 | 0.08 | 1.08 | 0.281 |
| Block [4] | 0.32 | 0.09 | 3.69 | **<0.001** |
| *Random Effects* | | | | |
| σ^2^ | 0.23 | | | |
| τ_00_ _Set_ | 0.00 | | | |
| N _Set_ | 16 | | | |
| Observations | 303 | | | |

**Table S4B.** Full model output of individual fitness for parents in experiment B.

| *Predictors* | *Estimates* | *std. Error* | *Statistic* | *p* |
| --- | --- | --- | --- | --- |
| (Intercept) | 2.82 | 0.05 | 55.77 | **<0.001** |
| Treatment [ev] | -0.07 | 0.05 | -1.59 | 0.113 |
| Block [2] | 0.05 | 0.06 | 0.80 | 0.421 |
| Block [3] | 0.40 | 0.06 | 6.27 | **<0.001** |
| Block [4] | -0.01 | 0.06 | -0.21 | 0.830 |
| *Random Effects* | | | | |
| σ^2^ | 0.16 | | | |
| τ_00_ _Set_ | 0.00 | | | |
| N _Set_ | 16 | | | |
| Observations | 316 | | | |

**Table S4C.** Full model output of individual fitness for parents in experiment C.

| *Predictors* | *Estimates* | *std. Error* | *Statistic* | *p* |
| --- | --- | --- | --- | --- |
| (Intercept) | 4.64 | 0.04 | 104.99 | **<0.001** |
| Treatment [ev] | 0.01 | 0.03 | 0.38 | 0.703 |
| Block [2] | -0.74 | 0.06 | -12.64 | **<0.001** |
| Block [3] | 0.03 | 0.06 | 0.56 | 0.577 |
| Block [4] | 0.17 | 0.06 | 2.88 | **0.004** |
| *Random Effects* | | | | |
| σ^2^ | 0.09 | | | |
| τ_00_ _Set_ | 0.00 | | | |
| N _Set_ | 16 | | | |
| Observations | 317 | | | |

**Table S4D.** Full model output of individual fitness for offspring in experiment A.

| *Predictors* | *Estimates* | *std. Error* | *Statistic* | *p* |
| --- | --- | --- | --- | --- |
| (Intercept) | 3.35 | 0.10 | 32.34 | **<0.001** |
| Treatment [ev] | -0.21 | 0.08 | -2.48 | **0.013** |
| Block [2] | -1.03 | 0.13 | -7.86 | **<0.001** |
| Block [3] | -3.03 | 0.14 | -21.16 | **<0.001** |
| Block [4] | -2.54 | 0.18 | -14.10 | **<0.001** |
| *Random Effects* | | | | |
| σ^2^ | 0.37 | | | |
| τ_00_ _Set_ | 0.01 | | | |
| N _Set_ | 16 | | | |
| Observations | 210 | | | |

**Table S4E.** Full model output of individual fitness for offspring in experiment B.

| *Predictors* | *Estimates* | *std. Error* | *Statistic* | *p* |
| --- | --- | --- | --- | --- |
| (Intercept) | 0.64 | 0.20 | 3.25 | **0.001** |
| Treatment [ev] | -0.03 | 0.10 | -0.24 | 0.810 |
| Block [2] | 1.23 | 0.25 | 4.84 | **<0.001** |
| Block [3] | 1.60 | 0.25 | 6.33 | **<0.001** |
| Block [4] | 0.11 | 0.27 | 0.40 | 0.692 |
| *Random Effects* | | | | |
| σ^2^ | 0.61 | | | |
| τ_00_ _Set_ | 0.08 | | | |
| N _Set_ | 16 | | | |
| Observations | 228 | | | |

**Table S4F.** Full model output of individual fitness for offspring in experiment C.

| *Predictors* | *Estimates* | *std. Error* | *Statistic* | *p* |
| --- | --- | --- | --- | --- |
| (Intercept) | 2.98 | 0.08 | 36.25 | **<0.001** |
| Treatment [ev] | -0.23 | 0.07 | -3.07 | **0.002** |
| Block [2] | 1.36 | 0.10 | 13.00 | **<0.001** |
| Block [3] | 0.78 | 0.10 | 7.48 | **<0.001** |
| Block [4] | 0.07 | 0.10 | 0.64 | 0.520 |
| *Random Effects* | | | | |
| σ^2^ | 0.42 | | | |
| τ_00_ _Set_ | 0.00 | | | |
| N _Set_ | 16 | | | |
| Observations | 308 | | | |

**Table S5A.** Full model output of health-span (speed) for parents in experiment A.

| *Predictors* | *Estimates* | *std. Error* | *Statistic* | *p* |
| --- | --- | --- | --- | --- |
| (Intercept) | 5.13 | 0.26 | 19.87 | **<0.001** |
| Seeding [ev] | 0.40 | 0.40 | 0.99 | 0.321 |
| Day | -0.19 | 0.03 | -6.57 | **<0.001** |
| Block [A3] | 0.36 | 0.16 | 2.19 | **0.028** |
| Seeding [ev] * Day | -0.19 | 0.05 | -3.76 | **<0.001** |
| *Random Effects* | | | | |
| σ^2^ | 1.25 | | | |
| τ_00_ _Plate_ | 0.00 | | | |
| τ_00_ _ID_ | 0.00 | | | |
| N _Plate_ | 12 | | | |
| N _ID_ | 104 | | | |
| Observations | 229 | | | |

**Table S5B.** Full model output of health-span (speed) for parents in experiment B.

| *Predictors* | *Estimates* | *std. Error* | *Statistic* | *p* |
| --- | --- | --- | --- | --- |
| (Intercept) | 4.85 | 0.26 | 18.77 | **<0.001** |
| Seeding [ev] | 0.64 | 0.45 | 1.43 | 0.152 |
| Day | -0.17 | 0.02 | -7.41 | **<0.001** |
| Block [B4] | 0.14 | 0.23 | 0.60 | 0.546 |
| Seeding [ev] * Day | -0.18 | 0.05 | -3.48 | **<0.001** |
| *Random Effects* | | | | |
| σ^2^ | 1.01 | | | |
| τ_00_ _Plate_ | 0.08 | | | |
| τ_00_ _ID_ | 0.00 | | | |
| N _Plate_ | 12 | | | |
| N _ID_ | 98 | | | |
| Observations | 224 | | | |

**Table S5C.** Full model output of health-span (speed) for parents in experiment C.

| *Predictors* | *Estimates* | *std. Error* | *Statistic* | *p* |
| --- | --- | --- | --- | --- |
| (Intercept) | 5.22 | 0.15 | 35.88 | **<0.001** |
| Seeding [ev] | -0.61 | 0.19 | -3.19 | **0.001** |
| Day | -0.32 | 0.02 | -20.95 | **<0.001** |
| Block [C2] | 0.83 | 0.14 | 5.81 | **<0.001** |
| Block [C3] | 0.94 | 0.14 | 6.48 | **<0.001** |
| Block [C4] | 0.92 | 0.15 | 6.29 | **<0.001** |
| Seeding [ev] * Day | 0.04 | 0.03 | 1.68 | 0.094 |
| *Random Effects* | | | | |
| σ^2^ | 0.66 | | | |
| τ_00_ _Plate_ | 0.03 | | | |
| τ_00 ID_ | 0.00 | | | |
| N _Plate_ | 24 | | | |
| N _ID_ | 220 | | | |
| Observations | 523 | | | |

**Table S5D.** Full model output of health-span (speed) for offspring in experiment A.

| *Predictors* | *Estimates* | *std. Error* | *Statistic* | *p* |
| --- | --- | --- | --- | --- |
| (Intercept) | 4.93 | 0.25 | 19.41 | **<0.001** |
| Seeding [ev] | 0.36 | 0.41 | 0.90 | 0.370 |
| Day | -0.28 | 0.04 | -7.08 | **<0.001** |
| Block [A3] | 0.01 | 0.18 | 0.06 | 0.956 |
| Seeding [ev] * Day | -0.04 | 0.06 | -0.57 | 0.567 |
| *Random Effects* | | | | |
| σ^2^ | 0.80 | | | |
| τ_00_ _Plate_ | 0.02 | | | |
| τ_00_ _ID_ | 0.00 | | | |
| N _Plate_ | 12 | | | |
| N _ID_ | 94 | | | |
| Observations | 166 | | | |

**Table S5E.** Full model output of health-span (speed) for offspring in experiment B.

| *Predictors* | *Estimates* | *std. Error* | *Statistic* | *p* |
| --- | --- | --- | --- | --- |
| (Intercept) | 5.14 | 0.31 | 16.76 | **<0.001** |
| Seeding [ev] | -0.25 | 0.43 | -0.60 | 0.551 |
| Day | -0.31 | 0.04 | -7.68 | **<0.001** |
| Block [B4] | -0.27 | 0.23 | -1.16 | 0.244 |
| Seeding [ev] * Day | 0.04 | 0.06 | 0.58 | 0.564 |
| *Random Effects* | | | | |
| σ^2^ | 0.95 | | | |
| τ_00_ _Plate_ | 0.09 | | | |
| τ_00_ _ID_ | 0.00 | | | |
| N _Plate_ | 12 | | | |
| N _ID_ | 100 | | | |
| Observations | 186 | | | |

**Table S5F.** Full model output of health-span (speed) for offspring in experiment C.

| *Predictors* | *Estimates* | *std. Error* | *Statistic* | *p* |
| --- | --- | --- | --- | --- |
| (Intercept) | 5.46 | 0.13 | 42.28 | **<0.001** |
| Seeding [ev] | -0.18 | 0.16 | -1.10 | 0.273 |
| Day | -0.38 | 0.02 | -20.19 | **<0.001** |
| Block [C2] | 0.74 | 0.10 | 7.33 | **<0.001** |
| Block [C3] | 0.91 | 0.10 | 8.72 | **<0.001** |
| Block [C4] | 0.80 | 0.10 | 8.12 | **<0.001** |
| Seeding [ev] * Day | 0.00 | 0.03 | 0.10 | 0.918 |
| *Random Effects* | | | | |
| σ^2^ | 0.61 | | | |
| τ_00_ _Plate_ | 0.00 | | | |
| τ_00_ _ID_ | 0.00 | | | |
| N _Plate_ | 24 | | | |
| N _ID_ | 232 | | | |
| Observations | 494 | | | |

**Table S6A.** Full model output of health-span (turns) for parents in experiment A.

**Poisson model w/ OLRE** – Poisson model w/o OLRE (AIC: **1076.7** – 1146.4)

| *Predictors* | *Log-Mean* | *std. Error* | *Statistic* | *p* |
| --- | --- | --- | --- | --- |
| (Intercept) | -1.43 | 0.18 | -8.11 | **<0.001** |
| Seeding [ev] | 0.21 | 0.28 | 0.73 | 0.463 |
| Day | -0.10 | 0.02 | -4.68 | **<0.001** |
| Block [A3] | 0.40 | 0.12 | 3.38 | **0.001** |
| Seeding [ev] * Day | -0.10 | 0.04 | -2.53 | **0.011** |
| *Random Effects* | | | | |
| σ^2^ | 0.61 | | | |
| τ_00_ _Plate_ | 0.00 | | | |
| τ_00_ _ID_ | 0.00 | | | |
| τ_00_ _obs_ | 0.34 | | | |
| N _Plate_ | 12 | | | |
| N _ID_ | 104 | | | |
| N _obs_ | 229 | | | |
| Observations | 229 | | | |

**Table S6B.** Full model output of health-span (turns) for parents in experiment B.

**Poisson model w/ OLRE** – Poisson model w/o OLRE (AIC: **996.4** – 1013.3)

| *Predictors* | *Log-Mean* | *std. Error* | *Statistic* | *p* |
| --- | --- | --- | --- | --- |
| (Intercept) | -1.79 | 0.14 | -12.42 | **<0.001** |
| Seeding [ev] | 0.66 | 0.28 | 2.38 | **0.017** |
| Day | -0.05 | 0.02 | -2.89 | **0.004** |
| Block [B4] | 0.16 | 0.12 | 1.34 | 0.181 |
| Seeding [ev] * Day | -0.17 | 0.04 | -4.41 | **<0.001** |
| *Random Effects* | | | | |
| σ^2^ | 0.45 | | | |
| τ_00_ _Plate2_ | 0.00 | | | |
| τ_00_ _ID_ | 0.06 | | | |
| τ_00_ _obs_ | 0.17 | | | |
| N _Plate2_ | 12 | | | |
| N _ID_ | 98 | | | |
| N _obs_ | 224 | | | |
| Observations | 224 | | | |

**Table S6C.** Full model output of health-span (turns) for parents in experiment C.

**Poisson model w/ OLRE** – Poisson model w/o OLRE (AIC: **2200.1** – 2218.7)

| *Predictors* | *Log-Mean* | *std. Error* | *Statistic* | *p* |
| --- | --- | --- | --- | --- |
| (Intercept) | -1.26 | 0.12 | -10.32 | **<0.001** |
| Seeding [ev] | -0.01 | 0.15 | -0.05 | 0.957 |
| Day | -0.19 | 0.01 | -14.46 | **<0.001** |
| Block [C2] | 0.34 | 0.13 | 2.65 | **0.008** |
| Block [C3] | 0.21 | 0.13 | 1.64 | 0.100 |
| Block [C4] | 0.44 | 0.13 | 3.43 | **0.001** |
| Seeding [ev] * Day | -0.01 | 0.02 | -0.54 | 0.589 |
| *Random Effects* | | | | |
| σ^2^ | 0.35 | | | |
| τ_00_ _Plate_ | 0.03 | | | |
| τ_00_ _ID_ | 0.00 | | | |
| τ_00_ _obs_ | 0.08 | | | |
| N _Plate_ | 24 | | | |
| N _ID_ | 220 | | | |
| N _obs_ | 523 | | | |
| Observations | 523 | | | |

**Table S6D.** Full model output of health-span (turns) for offspring in experiment A.

**Poisson model w/ OLRE** – Poisson model w/o OLRE (AIC: **818.5** – 863.3)

| *Predictors* | *Log-Mean* | *std. Error* | *Statistic* | *p* |
| --- | --- | --- | --- | --- |
| (Intercept) | -1.28 | 0.17 | -7.73 | **<0.001** |
| Seeding [ev] | -0.55 | 0.28 | -1.95 | 0.051 |
| Day | -0.17 | 0.03 | -5.85 | **<0.001** |
| Block [A3] | 0.74 | 0.11 | 6.50 | **<0.001** |
| Seeding [ev] * Day | 0.07 | 0.05 | 1.46 | 0.145 |
| *Random Effects* | | | | |
| σ^2^ | 0.41 | | | |
| τ_00_ _Plat_ | 0.00 | | | |
| τ_00 ID_ | 0.00 | | | |
| τ_00_ _obs_ | 0.20 | | | |
| N _Plate_ | 12 | | | |
| N _ID_ | 94 | | | |
| N _obs_ | 166 | | | |
| Observations | 166 | | | |

**Table S6E.** Full model output of health-span (turns) for offspring in experiment B.

**Compois model** – Poisson model w/o OLRE* (AIC: **800.8** – 802.7*)

*overdispersed

| *Predictors* | *Log-Mean* | *std. Error* | *Statistic* | *p* |
| --- | --- | --- | --- | --- |
| (Intercept) | -1.79 | 0.18 | -9.83 | **<0.001** |
| Seeding [ev] | 0.00 | 0.25 | 0.01 | 0.996 |
| Day | -0.12 | 0.03 | -4.00 | **<0.001** |
| Block [B4] | 0.53 | 0.12 | 4.47 | **<0.001** |
| Seeding [ev] * Day | -0.02 | 0.04 | -0.52 | 0.606 |
| *Random Effects* | | | | |
| σ^2^ | 1.40 | | | |
| τ_00_ _Plate_ | 0.01 | | | |
| τ_00_ _ID_ | 0.03 | | | |
| N _Plate_ | 12 | | | |
| N _ID_ | 100 | | | |
| Observations | 186 | | | |

**Table S6F.** Full model output of health-span (turns) for offspring in experiment C.

Poisson model w/ OLRE – **Poisson model w/o OLRE** (AIC: 2006.9 – **2006.0**)

| *Predictors* | *Log-Mean* | *std. Error* | *Statistic* | *p* |
| --- | --- | --- | --- | --- |
| (Intercept) | -1.00 | 0.12 | -8.14 | **<0.001** |
| Seeding [ev] | 0.02 | 0.13 | 0.16 | 0.870 |
| Day | -0.24 | 0.02 | -15.71 | **<0.001** |
| Block [C2] | 0.43 | 0.13 | 3.27 | **0.001** |
| Block [C3] | -0.10 | 0.13 | -0.73 | 0.464 |
| Block [C4] | 0.40 | 0.13 | 3.02 | **0.003** |
| Seeding [ev] * Day | -0.02 | 0.02 | -1.09 | 0.278 |
| *Random Effects* | | | | |
| σ^2^ | 0.22 | | | |
| τ_00_ _Plate_ | 0.04 | | | |
| τ_00_ _ID_ | 0.00 | | | |
| N _Plate_ | 24 | | | |
| N _ID_ | 232 | | | |
| Observations | 494 | | | |

**Table S7. Primer sequences**. Forward (fwd) and reverse (rev) sequences are listed in the 5’ to 3’ direction. Sequences acquired from [1] for *daf-2* and [2] for the *actin-3* reference gene.

| Gene | Primer Sequences |
| --- | --- |
| *daf-2* | Fwd: GTGGCGTGAGAATGAAGTGAG  Rev: GGCTTATCGGCTACAATCGTC |
| *actin-3* | Fwd: CCAAGAGAGGTATCCTTACCCTCAA  Rev: AAGCTCATTGTAGAAGGTGTGATGC |

**Table S8. Relative gene expression (ΔCt).** The effect of *daf-2* RNAi from the late-L4 stage on the expression of *daf-2* relative to the *actin-3* reference gene (ΔCt from qRT-PCR) at day 2 of adulthood. Treatment and qRT-PCR plate contrasts from linear model analysis of variance table.

| Factor | Sum Sq | Mean Sq | F | d.f. | p |
| --- | --- | --- | --- | --- | --- |
| RNAi treatment | 15.651 | 15.652 | 48.358 | 1 | **<0.001** |
| Plate | 0.970 | 0.485 | 1.499 | 2 | 0.233 |
| RNAi treatment x Plate | 0.516 | 0.285 | 0.797 | 2 | 0.456 |

**Figure S1.** Age-specific reproductive success (ARS) for parental and offspring generations across three experiments and four blocks (see Fig. 1 in the main MS legend for details).

**Figure S2.** Survival curves (with matricide) for parental and offspring generations across three experiments and four blocks (see Fig. 1 in the main MS legend for details).

**Figure S3.** Survival curves (without matricide) for parental and offspring generations across three experiments and four blocks (see Fig. 1 in the main MS legend for details).

**Figure S4.** Individual fitness for parental and offspring generations across all three experiments (see Fig. 1 in the main MS legend for details).

**Figure S4. Normalised *daf-2* expression following RNAi treatment versus untreated controls.** RNAi was delivered from the late-L4 stage and gene expression was quantified in two-day old adults using qRT-PCR, in individual worms (empty vector, ev, controls: n=29; *daf-2* RNAi: n=30; as separate points). Arithmetic mean of biological replicates shown as a red diamond with +/- 1 standard error bars, for each of the three qRT-PCR plates. Normalised *daf-2* expression (2 ^- ΔCT^) was calculated relative to expression of the *actin-3* reference gene [3].


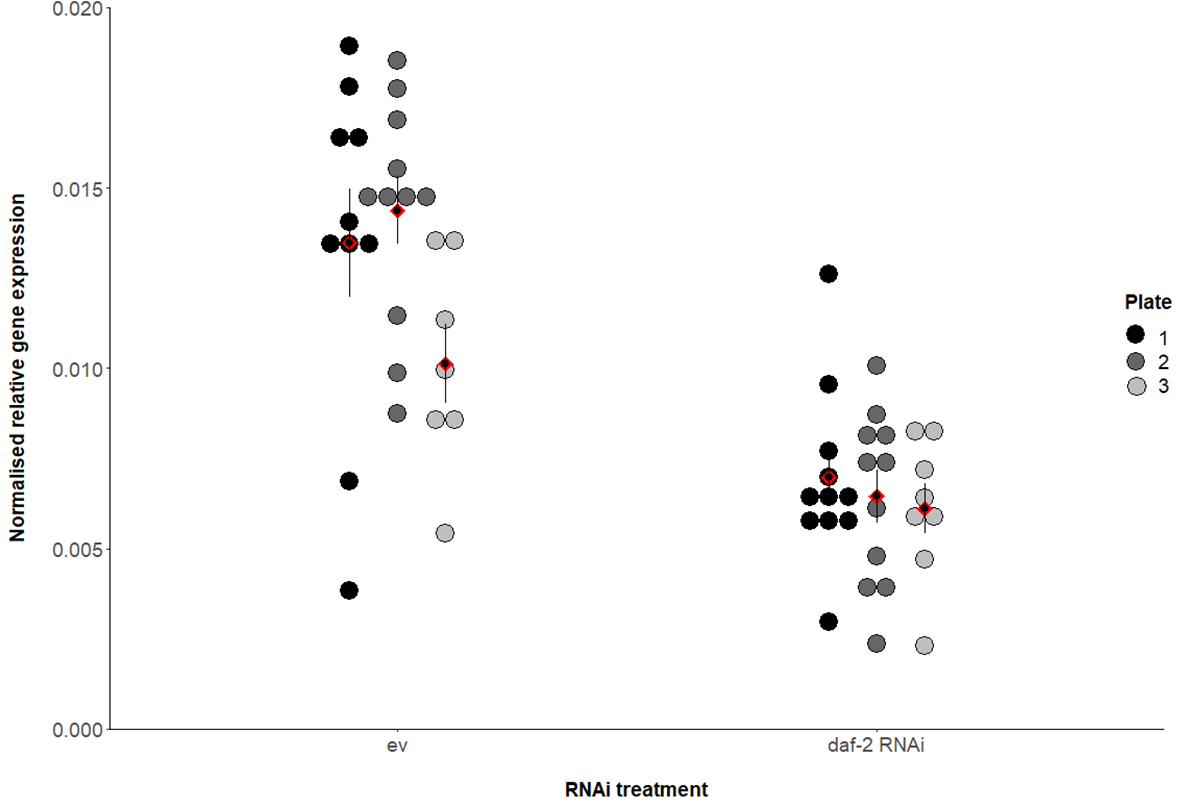


**References** (all references that appear here also appear in the main text)

1. Chauve, L., Le Pen, J., Hodge, F., Todtenhaupt, P., Biggins, L., Miska, E.A., Andrews, S., and Casanueva, O. (2020). High-Throughput Quantitative RT-PCR in Single and Bulk C. elegans Samples Using Nanofluidic Technology. Jove-Journal of Visualized Experiments.

2. Akay, A., Di Domenico, T., Suen, K.M., Nabih, A., Parada, G.E., Larance, M., Medhi, R., Berkyurek, A.C., Zhang, X.L., Wedeles, C.J., et al. (2017). The Helicase Aquarius/EMB-4 Is Required to Overcome Intronic Barriers to Allow Nuclear RNAi Pathways to Heritably Silence Transcription. Developmental Cell *42*, 241-+.

3. Schmittgen, T.D., and Livak, K.J. (2008). Analyzing real-time PCR data by the comparative C-T method. Nature Protocols *3*, 1101-1108.
